# Proteasome alteration between epithelial and hematopoietic cells facilitates positive selection of CD8 T cells

**DOI:** 10.1038/s41467-026-72411-x

**Published:** 2026-04-27

**Authors:** Mami Matsuda-Lennikov, Jamie-Jean De La Torre, Todd Snow, Jacqueline Battaile, Felix Kalle-Youngoue, Alison Jacques, Aya Ushio, Akihide Shimizu, Miho Shinzawa, Christian T. Mayer, Parirokh Awasthi, Raj Chari, Izumi Ohigashi, Shigeo Murata, Yousuke Takahama

**Affiliations:** 1 Thymus Biology Section, Experimental Immunology Branch, Center for Cancer Research, National Cancer Institute, National Institutes of Health, Bethesda, MD USA; 2 Laboratory Animal Sciences Program, Frederick National Laboratory for Cancer Research, National Cancer Institute, National Institutes of Health, Frederick, MD USA; 3 Lymphocyte Development Section, Experimental Immunology Branch, Center for Cancer Research, National Cancer Institute, National Institutes of Health, Bethesda, MD USA; 4 Immunomodulation Unit, Experimental Immunology Branch, Center for Cancer Research, National Cancer Institute, National Institutes of Health, Bethesda, MD USA; 5 Mouse Modeling and Cryopreservation, Laboratory Animal Sciences Program, Frederick National Laboratory for Cancer Research, National Cancer Institute, National Institutes of Health, Frederick, MD USA; 6 Genome Modification Core, Laboratory Animal Sciences Program, Frederick National Laboratory for Cancer Research, National Cancer Institute, National Institutes of Health, Frederick, MD USA; 7 Division of Experimental Immunology, Institute of Advanced Medical Sciences, Tokushima University, Tokushima, Japan; 8 Laboratory of Protein Metabolism, Graduate School of Pharmaceutical Sciences, University of Tokyo, Bunkyo, Japan

**Keywords:** Clonal selection, Thymus

## Abstract

The thymoproteasome, a proteolytic complex uniquely expressed in cortical thymic epithelial cells (cTECs), governs the positive selection of CD8 T cells. It has been hypothesized that the thymoproteasome promotes CD8 T cell development by forging a sequential switch in proteasome species between cTECs and medullary antigen-presenting cells (APCs), which generates a stepwise difference in MHC-I-associated peptides between cTECs and medullary APCs, thereby sparing positively selected thymocytes from subsequent negative selection. In this study, we engineer mice ectopically expressing thymoproteasomes in various APCs including medullary TECs (mTECs), eliminating the proposed proteasome switch. Surprisingly, we find that the proteasome switch between cTECs and mTECs is dispensable for thymoproteasome-dependent CD8 T cell development. Instead, we find that ectopic thymoproteasomes in hematopoietic cells impair CD8 T cell development by hindering cortical positive selection. Our findings reveal that the proteasome difference between cTECs and hematopoietic cells in the thymic cortex facilitates thymoproteasome-dependent positive selection.

## Abstract (web summary)

Thymoproteasomes expressed in cortical thymic epithelial cells are essential for the generation of CD8^+^ T cells, but the mechanism requires further exploration. By engineering a mouse strain ectopically expressing thymoproteasomes in various antigen-presenting cells including medullary TECs, the authors identify that thymoproteasomes specifically expressed in cortical TECs but not hematopoietic cells facilitate positive selection of CD8^+^ T cells.

## Introduction

Immature thymocytes that express a germline repertoire of TCR-αβ antigen-receptor complexes arise in the thymic cortex and are screened for their capability for TCR interactions with self-peptide-MHC complexes provided by cortical thymic epithelial cells (cTECs)^1,2^. This process of cortical positive selection enriches T cells that are capable of interacting at low affinity with self-peptide-MHC complexes, contributing to the formation of a mature T cell repertoire that is reactive at high affinity with MHC-associated foreign antigen peptides displayed by peripheral antigen-presenting cells (APCs), including dendritic cells (DCs) in secondary lymphoid organs. Interestingly, cTECs possess unique protein degradation machinery for processing MHC-associated self-peptides that promote T-cell positive selection^2,3^. To optimize MHC-I-restricted positive selection of CD8^+^ T cells, cTECs uniquely express proteasomal subunit β5t encoded by *Psmb11*^4^. β5t is a proteolytic component assembled in the proteasome complex to form the cTEC-specific thymoproteasome^4,5^. The β5t-containing thymoproteasome promotes cTEC-specific protein degradation and processing of MHC-I-associated self-peptides, which optimizes positive selection of CD8^+^ T cells^4–10^.

How the thymoproteasome-dependent MHC-I-associated peptides contribute to CD8^+^ T-cell positive selection is controversial. It is hypothesized that forging a stepwise difference in proteasome species between positively selecting cTECs and negatively selecting medullary APCs is the key to the function of thymoproteasomes uniquely expressed by cTECs^11,12^. Unlike cTECs expressing β5t-containing thymoproteasome, all other thymic APCs, including medullary TECs (mTECs), DCs, and B cells, predominantly express the immunoproteasome and its component β5i instead of thymoproteasome-specific β5t component^4,5,13^. The sequential switch in proteasome species between cTECs and medullary APCs is believed to generate the difference in MHC-I-associated self-peptides between positively selecting cTECs and negatively selecting medullary APCs. It is predicted that the difference in MHC-I-associated self-peptides would create a window for positively selected CD8-lineage thymocytes to escape from the subsequent negative selection induced by thymic medullary APCs. Consequently, the β5t-dependent “proteasome switch” between cTECs and medullary APCs would, in turn, optimize the generation of an optimized pool of self-protective naïve CD8^+^ T cells^11,12^. Alternatively, but not mutually exclusively, it is also possible that thymoproteasome-dependent MHC-I-associated peptides have a yet-unidentified advantage in inducing the positive selection of CD8-lineage thymocytes in the thymic cortex^6–8^. In this context, it is important to note that the thymoproteasome in cTECs optimizes CD8^+^ T cell development even in the absence of MHC-I presentation by other thymic APCs, including mTECs, DCs, and B cells^14,15^, indicating that the thymoproteasome mediates CD8^+^ T-cell positive selection even without the contribution of negatively selecting APCs in the thymic medulla. In addition, a previously unknown mechanism may contribute to the thymoproteasome-dependent CD8^+^ T cell development.

The present study was undertaken to directly address the β5t-dependent proteasome switch hypothesis. We generated mice genetically engineered to ectopically express the thymoproteasome instead of the immunoproteasome. The thymoproteasome, expressed specifically in cTECs, and the immunoproteasome, expressed in other thymic APCs including mTECs, DCs, and B cells, differ only in the catalytic subunit β5t or β5i, while sharing all other proteasome subunits^4,5,16^. We report here that the newly engineered animals ectopically express the thymoproteasome widely in thymic APCs, including mTECs, DCs, and B cells, thereby canceling out the proteasome switch between cTECs and other thymic APCs. Our results indicate that the proteasome switch between cTECs and mTECs is dispensable for thymoproteasome-dependent development of CD8^+^ T cells. Unexpectedly, we find that ectopically thymoproteasome-expressing hematopoietic cells hinder endogenous thymoproteasome-dependent positive selection of CD8^+^ T cells. We further demonstrate that the β5t-dependent alteration of proteasome species between cTECs and hematopoietic cells facilitates thymoproteasome-mediated positive selection in the thymic cortex. These results provide novel insight into the mechanism underlying the thymoproteasome-dependent positive selection of CD8^+^ T cells by highlighting the role of the thymoproteasome in securing the unique capability of cTECs, rather than cortical hematopoietic APCs, to induce positive selection in the thymic cortex.

## Results

### Ectopic expression of thymoproteasomes instead of immunoproteasomes in BIT mice

**Fig. 1:**
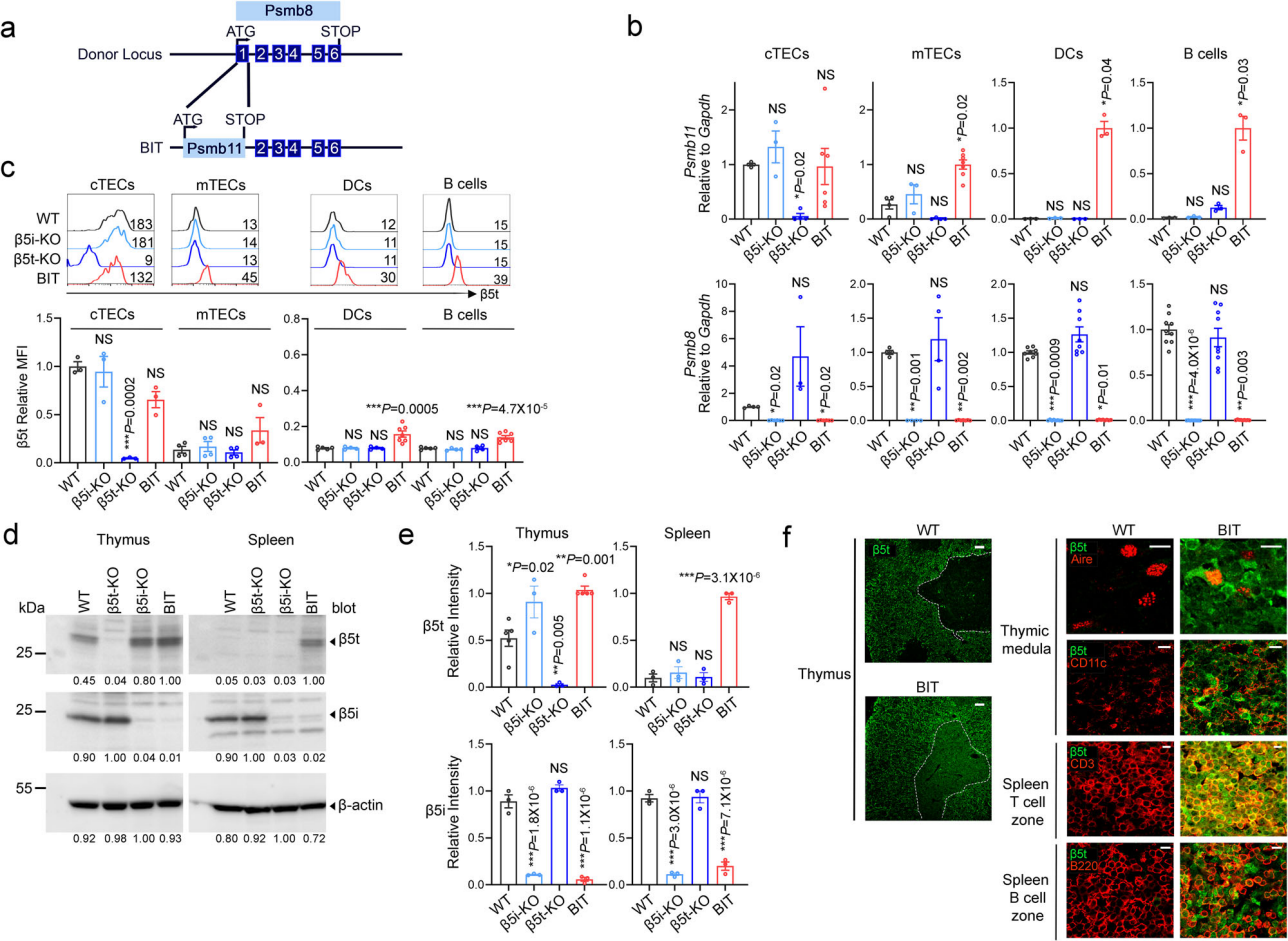
Ectopic expression of β5t in BIT mice. **a** Scheme for engineering β5i^β5t^ (BIT) allele. β5t-encoding *Psmb11* sequence was inserted at the translation initiation site within the exons 1 of β5i-encoding *Psmb8* gene in the mouse genome. **b** Indicated cells from the thymus of WT, β5i-KO, β5t-KO, and BIT mice were sorted by flow cytometry. *Psmb11* and *Psmb8* mRNAs were measured by quantitative RT-PCR analysis and normalized to *Gapdh* mRNAs. Data from are presented as means ± SEMs. *Psmb11*; cTECs (WT, *n* = 4; β5i-KO, *n* = 3; β5t-KO, *n* = 4; BIT, *n* = 6), mTECs (WT, *n* = 4; β5i-KO, *n* = 3; β5t-KO, *n* = 4; BIT, *n* = 7), DCs (WT, *n* = 3; β5i-KO, *n* = 3; β5t-KO, *n* = 3; BIT, *n* = 3), and B cells (WT, *n* = 3; β5i-KO, *n* = 3; β5t-KO, *n* = 3; BIT, *n* = 3). *Psmb8*; cTECs (WT, *n* = 4; β5i-KO, *n* = 6; β5t-KO, *n* = 3; BIT, *n* = 4), mTECs (WT, *n* = 4; β5i-KO, *n* = 4; β5t-KO, *n* = 4; BIT, *n* = 7), DCs (WT, *n* = 8; β5i-KO, *n* = 8; β5t-KO, *n* = 8; BIT, *n* = 4), and B cells (WT, *n* = 9; β5i-KO, *n* = 9; β5t-KO, *n* = 9; BIT, *n* = 9). Data from three independent experiments are shown. Statistical analysis was performed using the Kruskal–Wallis test followed by Dunn’s multiple comparisons test. *P < 0.05; **P < 0.01; ***P < 0.001; NS, not significant. **c** Flow cytometric analysis of intracellular β5t expression in indicated cells from the thymus of WT, β5t-KO, β5t-KO, and BIT mice. cTECs (WT, *n* = 3; β5i-KO, *n* = 3; β5t-KO, *n* = 3; BIT, *n* = 3), mTECs (WT, *n* = 4; β5i-KO, *n* = 4; β5t-KO, *n* = 4; BIT, *n* = 3), DCs (WT, *n* = 5; β5i-KO *n* = 4; β5t-KO, *n* = 4; BIT, *n* = 7) and B cells (WT, *n* = 5; β5i-KO, *n* = 4; β5t-KO, *n* = 4; BIT, *n* = 7). Shown are representative histograms (top) and relative mean fluorescence intensities (MFIs) (bottom). Data from three independent experiments are presented as means ± SEMs. Statistical analysis was performed using one-way ANOVA followed by Dunnett’s multiple comparisons test (each group compared with the WT group). **d** Lysates of the thymus and spleen from the indicated mice were subjected to immunoblot analysis with the indicated antibodies. β-actin was analyzed as a loading control. Representative blots from three independent experiments with WT, β5i-KO, β5t-KO, and BIT are shown. β5t blot thymus, WT (*n* = 5), β5i-KO (*n* = 3), β5t-KO (*n* = 3), BIT (*n* = 5). β5t blot spleen, WT (*n* = 3), β5i-KO (*n* = 3), β5t-KO (*n* = 3), BIT (*n* = 3). β5i blot thymus and spleen (WT, *n* = 3; β5i-KO, *n* = 3; β5t-KO, *n* = 3; BIT, *n* = 3). **e** Bar graphs represent the means ± SEMs of the chemiluminescence signals measured in (**d**). *P < 0.05, **P < 0.01, ***P < 0.001, NS, not significant. Statistical analysis was performed using one-way ANOVA followed by Dunnett’s multiple comparisons test (each group compared with the WT group). **f** Tissue sections from indicated mice were analyzed for β5t expression (green) and Aire (red), CD11c (red), CD3 (red), or B220 (red). Scale bars, 100 µm (β5t single color images in the left); 10 µm (two color images in the right). Representative images from six independent experiments are shown. Dashed lines, corticomedullary boundaries in the thymus sections.

To examine the contribution of the corticomedullary proteasome switch to thymoproteasome-dependent CD8^+^ T cell development, we engineered the genome of C57BL/6 mice to replace the β5i-encoding *Psmb8* sequence with the β5t-encoding *Psmb11* sequence (Fig. 1a). The resultant allele, termed β5i^β5t^, or BIT, was designed to produce β5t instead of β5i. Thymoproteasomes and immunoproteasomes differ only in the proteolytic subunit β5t or β5i, assembled in the β-ring sharing β1i, β2i, β3, β4, β6, and β7 subunits of 20S proteasome core particles^5,16^, so that mice carrying the homozygous BIT allele, or BIT mice, would ectopically express thymoproteasomes in cells that otherwise express immunoproteasomes. Indeed, β5t-encoding *Psmb11* mRNAs were detected in thymic APCs, including cTECs, mTECs, DCs, and B cells, in BIT mice (Fig. 1b). In contrast, *Psmb11* mRNAs were detected specifically in cTECs rather than other thymic APCs in wildtype (WT) mice (Fig. 1b). Furthermore, β5i-encoding *Psmb8* mRNAs detected in these thymic APCs in WT mice were lost in BIT mice (Fig. 1b). Along with the detection of *Psmb11* mRNAs, β5t proteins were ectopically detected in all these thymic APCs, unlike cTEC-specific expression in WT mice (Fig. 1c). β5t proteins were detected even in the spleen of BIT mice, whereas β5i proteins were lost in the thymus and spleen of BIT mice (Fig. 1d, e). Immunofluorescence analysis reconfirmed that BIT mice ectopically expressed β5t proteins in the thymic medulla, including Aire^+^ mTECs and thymic DCs, as well as in the spleen, including B cells and T cells (Fig. 1f). These results demonstrate the ectopic expression of β5t-containing thymoproteasomes in BIT mice and the cancellation of the β5t-dependent proteasome switch between their thymic microenvironments.

We engineered two different genomic modifications in the *Psmb8* allele to produce six independent BIT mouse lines (Supplementary Fig. 1a). All mouse lines exhibited essentially identical phenotypes in ectopic β5t expression as well as in CD8^+^ T cell development, as detailed below (Supplementary Fig. 1b, c). Hereinafter, we document the results from one BIT mouse line (BIT #1–2), unless otherwise indicated.

### Moderate reduction of CD8^+^ T cells in BIT mice

**Fig. 2:**
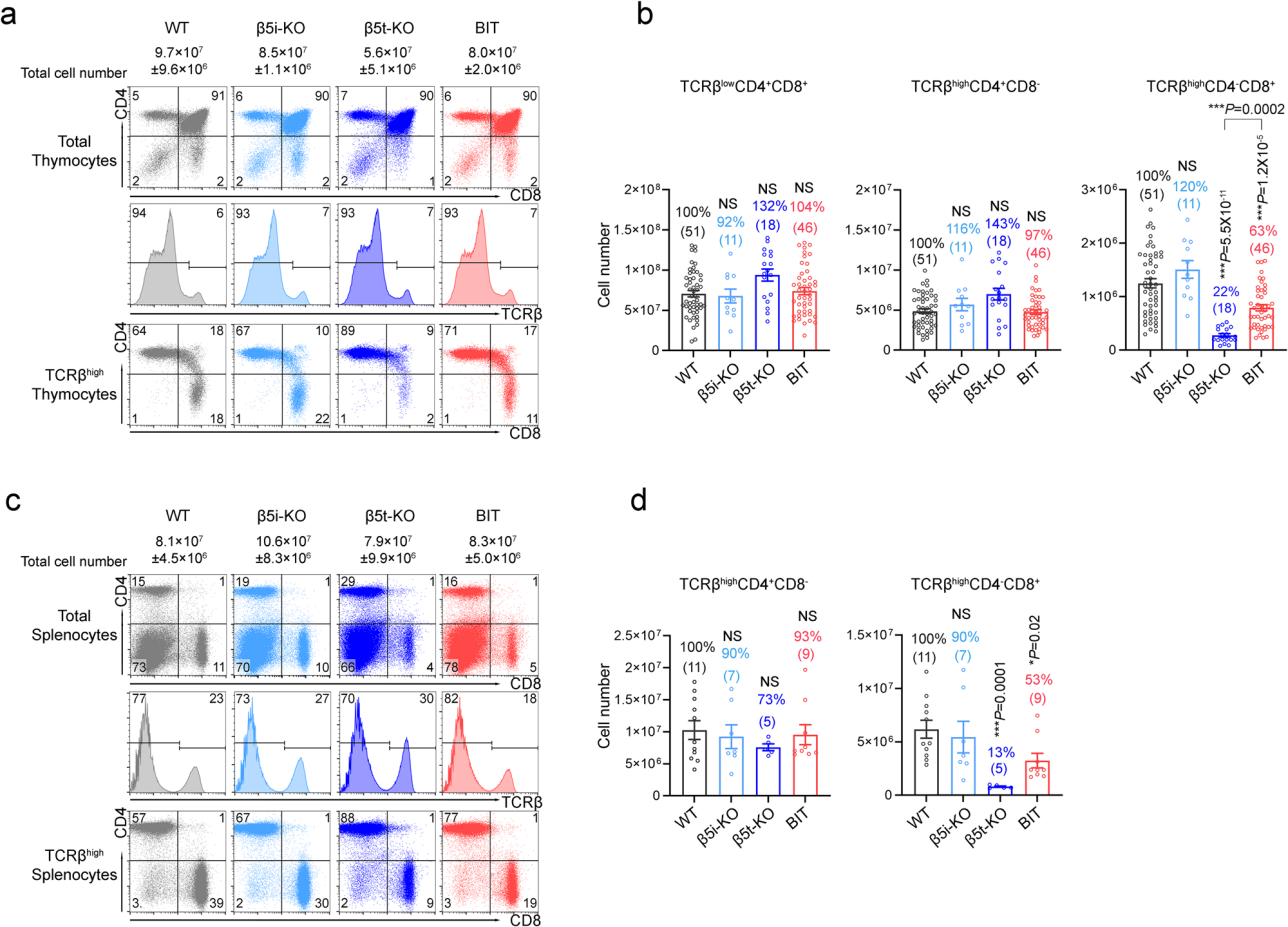
Moderate decrease of CD8⁺ T cells in BIT mice. Representative flow cytometric profiles of thymocytes (**a**) and splenocytes (**c**) from WT, β5i-KO, β5t-KO, and BIT mice (13-20 independent experiments). Cells were surface-stained for CD4, CD8, and TCRβ. Numbers in boxes indicate the percentage of cells in the indicated areas. Means ± standard errors of total cell numbers are also shown. Absolute numbers (per mouse) of indicated populations of thymocytes (**b**) and splenocytes (**d**) from WT, β5i-KO, β5t-KO, and BIT mice at 4 to 6 weeks old. **b** WT (*n* = 51), β5i-KO (*n* = 11), β5t-KO (*n* = 18), BIT (*n* = 46). **d** WT (*n* = 11), β5i-KO (*n* = 7), β5t-KO (*n* = 5), BIT (*n* = 9). Bar graphs represent the means ± SEMs. *P < 0.05, ***P < 0.001, NS, not significant. Statistical analysis was performed using one-way ANOVA followed by Šidák’s multiple comparisons test.

To examine T cell development in BIT mice, thymocytes and splenocytes from BIT mice, as well as from β5i-deficient mice and β5t-deficient mice, were analyzed for their cell-surface expression of CD4, CD8, and TCRβ. As previously reported^4,7,8^, β5t-deficient mice exhibited severe impairment in CD8^+^ T cell development, as the numbers of CD4^−^CD8^+^ TCR^high^ cells in the thymus and the spleen of β5t-deficient mice were 13–22% of the numbers in WT mice (Fig. 2a–d). In contrast, β5i-deficient mice generated a normal number of CD8^+^ T cells (Fig. 2a–d), indicating that the loss of β5i does not diminish CD8^+^ T cell development. Interestingly, however, the numbers of CD8^+^ T cells in the thymus and the spleen of BIT mice, in which β5t was ectopically expressed instead of β5i, were 53–63% of the numbers in WT mice (Fig. 2a–d). The relatively moderate reduction of CD8^+^ T cells in BIT mice was reproduced equivalently in all six BIT mouse lines engineered in this study (Supplementary Fig. 1b, c), unlike the severe reduction of CD8^+^ T cells in all four lines of β5t-deficient mouse strains reported so far^4,8,17,18^ (as exemplified in Fig. 2 and Supplementary Fig. 1b, c). None of these mouse lines exhibited impairment in CD4^+^ T cell development (Fig. 2, Supplementary Fig. 1b, c)^4,7,8,17,18^. These results indicate that the ectopic expression of β5t-containing thymoproteasomes instead of β5i-containing immunoproteasomes modestly impairs CD8^+^ T cell development, unlike the severe impairment of CD8^+^ T cell development in the absence of thymoproteasomes. Thus, the loss of β5t-dependent proteasome switch in BIT mice does not fully phenocopy the severe impairment of CD8^+^ T cell development by the loss of β5t, so that the β5t-dependent proteasome switch cannot fully explain the *raison d’etre* of the thymoproteasome.

It was previously reported that the genetic loss of β5i resulted in a decrease in MHC-I expression in various APCs, even though this decrease in MHC-I was not accompanied by a reduction of CD8^+^ T cells^19^. Accordingly, we noted that the expression of surface MHC-I H-2K^b^ and H-2D^b^ molecules was reduced (59–87% of WT values in the relative fluorescence intensity) in mTECs, DCs, and B cells from the thymus of β5i-deficient mice (Supplementary Fig. 2), whereas CD8^+^ T cell development was not reduced in these mice (Fig. 2a–d, Supplementary Fig. 1b, c). The reduction of MHC-I expression in β5i-deficient mice was minor and not statistically significant in cTECs (85–87% of WT values in the relative fluorescence intensity) in comparison with that in other APCs (59–81% of WT values) (Supplementary Fig. 2). We also noted that the slightly reduced MHC-I expression due to the absence of β5i was restored in BIT mice by the ectopic expression of β5t in cTECs but not in other APCs (Supplementary Fig. 2). These differences between cTECs and other cells possibly reflected abundant presence of β5t specifically in cTECs and different expression of other constitutive and immuno-proteasome subunits, which differently affected the assembly of functional proteasome particles^5,16,20^. Indeed, cTECs were low in the expression of constitutive β1 and β5 subunits in comparison with other cells, including mTECs^21^. Nevertheless, these results indicate that the moderately reduced CD8^+^ T cell development in BIT mice is not due to the reduction in MHC-I expression in positive selection-inducing cTECs.

### Proteasome switch between cTECs and mTECs is dispensable for CD8^+^ T cell development

**Fig. 3:**
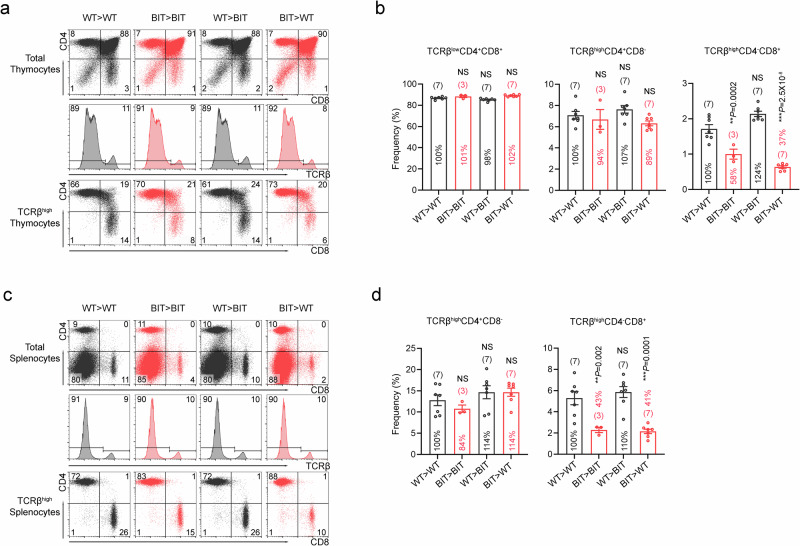
CD8⁺ T cell development in BIT bone marrow chimeric mice. Representative flow cytometric profiles of thymocytes (**a**) and splenocytes (**c**) from bone marrow chimeric mice reconstituting WT or BIT bone marrow donor cells into WT or BIT irradiated recipient mice as indicated (5 independent experiments). Numbers in boxes indicate the percentage of cells in the indicated areas. Frequency of indicated populations of thymocytes (**b**) and splenocytes (**d**) from WT > WT (*n* = 7), BIT > BIT (*n* = 3), WT > BIT (*n* = 7), and BIT > WT (*n* = 7) bone marrow chimeric mice six to eight weeks after the reconstitution. Data are presented as means ± SEMs. *P < 0.05, **P < 0.01, ***P < 0.001, NS, not significant. Statistical analysis was performed using one-way ANOVA followed by Šidák’s multiple comparisons test.

To begin exploring the mechanism for the moderate reduction of CD8^+^ T cells in BIT mice, we generated bone marrow chimeras in which either BIT mice or WT mice were reciprocally used as the source of bone marrow donor hematopoietic stem cells and/or lethally irradiated recipient mice. Bone marrow donor-derived cells and recipient-derived radioresistant cells were distinguished using mice carrying either CD45.1 or CD45.2 allelic variants. As expected, CD8^+^ T cells were moderately but significantly reduced (43–58%) in the thymus and the spleen of BIT recipient mice reconstituted with BIT bone marrow-derived cells (BIT > BIT), in comparison with CD8^+^ T cells generated in control bone marrow chimeras (WT > WT) (Fig. 3). Interestingly, however, WT hematopoietic stem cell-derived CD8^+^ T cells generated in the thymic microenvironment of BIT mice (WT > BIT) were comparable to, and not at all reduced in comparison with, CD8^+^ T cells generated in WT control bone marrow chimeras (WT > WT) (Fig. 3). These results indicate that non-hematopoietic thymic microenvironment in BIT mice is fully capable of promoting CD8^+^ T cell development, with its capability equivalent to the thymic environment in control WT mice. The β5t-dependent proteasome switch is lost between cTECs and mTECs in BIT mice (Fig. 1), so that the absence of compromise in CD8^+^ T cell development in WT > BIT bone marrow chimeras indicates that the proteasome switch between cTECs and mTECs is dispensable for thymoproteasome-dependent optimal development of CD8^+^ T cells.

### Hematopoietic MHC-I-expressing cells hinder CD8^+^ T cell development in BIT mice

In contrast to WT > BIT bone marrow chimeric mice, chimeric mice in which BIT mouse-derived hematopoietic cells developed in the non-hematopoietic microenvironment of WT mice (BIT > WT) showed a moderate reduction of CD8^+^ T cells (Fig. 3a–d), just like the modest reduction in BIT mice and BIT > BIT bone marrow chimeras. Thus, hematopoietic cells, rather than non-hematopoietic environments, were responsible for the moderate reduction of CD8^+^ T cells in BIT mice.

**Fig. 4:**
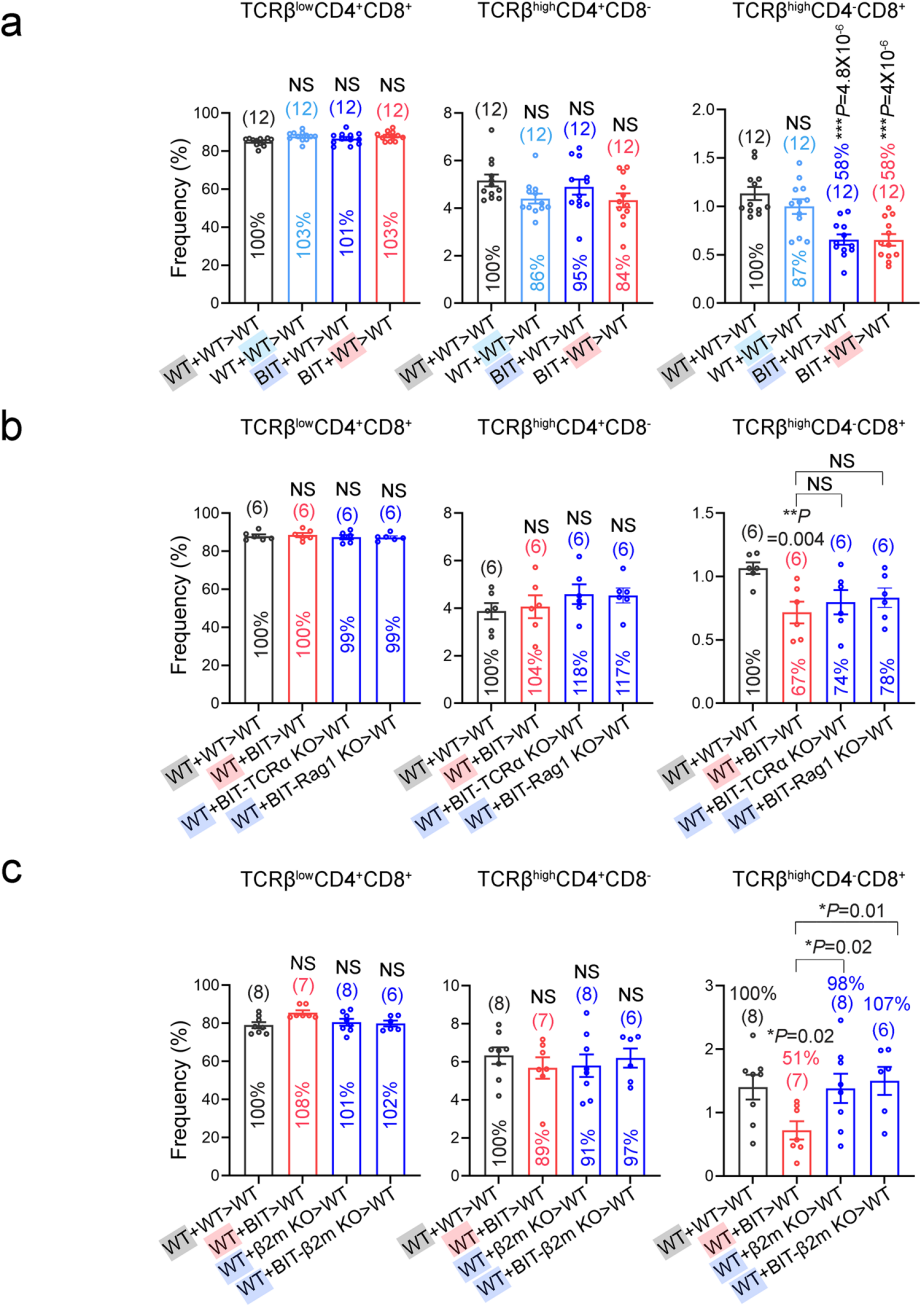
CD8⁺ T cell development in mixed bone marrow chimeric mice. **a** T cell-depleted bone marrow cells from B6 (WT) or BIT mice were 1:1 mixed with T cell-depleted bone marrow cells from B6-CD45.1 (WT) mice. Mixed bone marrow cells were transferred into lethally irradiated B6 × B6-CD45.1 F1 (WT) mice. WT + WT > WT (*n* = 12), WT + WT > WT (*n* = 12), BIT + WT > WT (*n* = 12), and BIT + WT > WT (*n* = 12). **b** T cell-depleted bone marrow cells from B6-CD45.1 (WT) mice were 1:1 mixed with T cell-depleted bone marrow cells from B6 (WT), BIT, TCRα-deficient BIT, or Rag1-deficient BIT mice. Mixed bone marrow cells were transferred into lethally irradiated B6 × B6-CD45.1 F1 (WT) mice. WT + WT > WT (*n* = 6), WT + BIT > WT (*n* = 6), WT + BIT-TCRαKO > WT (*n* = 6), and WT + BIT-Rag1KO>WT (*n* = 6). **c** T cell-depleted bone marrow cells from B6-CD45.1 (WT) mice were 1:1 mixed with T cell-depleted bone marrow cells from B6 (WT), BIT, β2m-deficient B6, or β2m-deficient BIT mice. Mixed bone marrow cells were transferred into lethally irradiated B6 x B6-CD45.1 F1 (WT) mice. WT + WT > WT (*n* = 8), WT + BIT > WT (*n* = 7), WT + β2mKO > WT (*n* = 8), and WT + BIT-β2mKO > WT (*n* = 6). Cells were distinguished by using monoclonal antibodies specific for CD45.1 and CD45.2. Six to eight weeks after the reconstitution, thymocytes were analyzed by flow cytometry for CD4, CD8 and TCRβ surface expression. Graphs indicate percentages of indicated populations in color-highlighted bone marrow origins (grey, blue, or red). Numbers in parentheses indicate sample numbers. Data were obtained from two to five independent experiments. Data are presented as means ± SEMs. * *P* < 0.05, ***P < 0.001, NS, not significant. Statistical analysis was performed using one-way ANOVA followed by Šidák’s multiple comparisons test.

To examine whether this impairment was intrinsic to developing T cells or due to interference from bystander hematopoietic cells, we generated mixed bone marrow chimeric mice in which BIT hematopoietic cells (CD45.2) and WT hematopoietic cells (CD45.1) would develop together at a 1:1 ratio in a radioresistant non-hematopoietic environment in irradiated WT host mice (CD45.1/2) ([BIT + WT] > WT). As in BIT > WT chimeric mice, we found that BIT mouse-derived hematopoietic stem cells in [BIT + WT] > WT chimeric mice gave rise to a moderate reduction of CD8^+^ T cells even in the presence of WT-derived T cell development, in comparison with WT hematopoietic stem cell-derived CD8^+^ T cells generated in control [WT + WT] > WT chimeras (Fig. 4a). More interestingly, WT bone marrow-derived hematopoietic stem cells in [BIT + WT] > WT chimeras exhibited a moderate reduction of CD8^+^ T cells, similar to the compromised CD8^+^ T cell development observed in co-developing BIT bone marrow-derived cells (Fig. 4a). These results indicate that bystander BIT hematopoietic cells hinder CD8^+^ T cell development derived from normal hematopoietic stem cells in the normal thymic environment. These results also indicate that the copresence of normal hematopoietic stem cell-derived thymocyte development does not restore the moderately reduced CD8^+^ T cell development derived from BIT hematopoietic stem cells.

To better understand the mechanism of this hindrance by BIT-derived hematopoietic cells, we examined CD8^+^ T cell development from WT hematopoietic stem cells in bone marrow chimeric mice in the presence of hematopoietic cells prepared from either TCRα-deficient BIT mice, which lacked TCRαβ-expressing cells, or Rag1-deficient BIT mice, which lacked T and B cells (Fig. 4b). In these [WT + BIT-TCRα-KO] > WT and [WT + BIT-Rag1-KO] > WT bone marrow chimeras, WT hematopoietic stem cell-derived CD8^+^ T cell development in the thymus was moderately reduced in the presence of bystander hematopoietic cells derived from either TCRα-deficient BIT mice or Rag1-deficient BIT mice, similarly to that in the presence of BIT hematopoietic cells (no statistical difference among the three groups, Fig. 4b). These results indicate that thymoproteasome-expressing BIT hematopoietic cells hinder CD8^+^ T cell development even when the interfering BIT cells are devoid of TCRαβ-expressing cells or limited to non-T non-B hematopoietic cells including DCs.

BIT-derived hematopoietic cells hindered CD8^+^ T cell development but not CD4^+^ T cell development in the thymus (Figs. 2, 3, and 4a, b), indicating that the hindrance is specific for the MHC-I-restricted CD8-lineage but not the MHC-II-restricted CD4-lineage of thymocyte development. MHC-I expression is detectable in a variety of hematopoietic cells, including DCs and B cells (Supplementary Fig. 2), even in immature CD4^ + ^CD8^+^ thymocytes at a low level^22,23^ (Supplementary Fig. 3). We next examined whether MHC-I molecules expressed by BIT hematopoietic cells were responsible for the CD8-lineage-specific hindrance of T cell development in BIT mice. Analysis of WT hematopoietic stem cell-derived CD8^+^ T cell development in [WT + BIT-β2m-KO] > WT mixed bone marrow chimeric mice showed that the loss of cell surface MHC-I expression due to the lack of β2m in BIT hematopoietic cells nullified the hindrance of WT hematopoietic stem cell-derived CD8^+^ T cell development, unlike WT cell-derived CD8^+^ T cell development that was compromised in [WT + BIT] > WT bone marrow chimeric mice (Fig. 4c). These results demonstrate that BIT hindrance of CD8^+^ T cell development is dependent on MHC-I molecules expressed by thymoproteasome-expressing BIT hematopoietic cells, indicating that ectopic thymoproteasome-expressing hematopoietic cells interfere with CD8^+^ T cell development via MHC-I-dependent presentation of thymoproteasome-dependent self-peptides.

### CD8^+^ T cell development in BIT mice is hindered in the thymic cortex

We next sought to determine the developmental stage of thymocytes at which ectopically thymoproteasome-expressing BIT hematopoietic cells hindered CD8^+^ T cell development. To this end, we examined BIT thymocyte development without the influence of MHC-II-dependent selection and development of CD4-lineage T cells by engineering BIT > I-A^b^-KO bone marrow chimeric mice. The genomic proximity between the β5i-encoding *Psmb8* locus, including the BIT allele, and the I-A-encoding *H2-Ab1* locus within the *H-2* region of mouse chromosome 17 did not allow us to successfully obtain I-A-deficient BIT mice in this study.

**Fig. 5:**
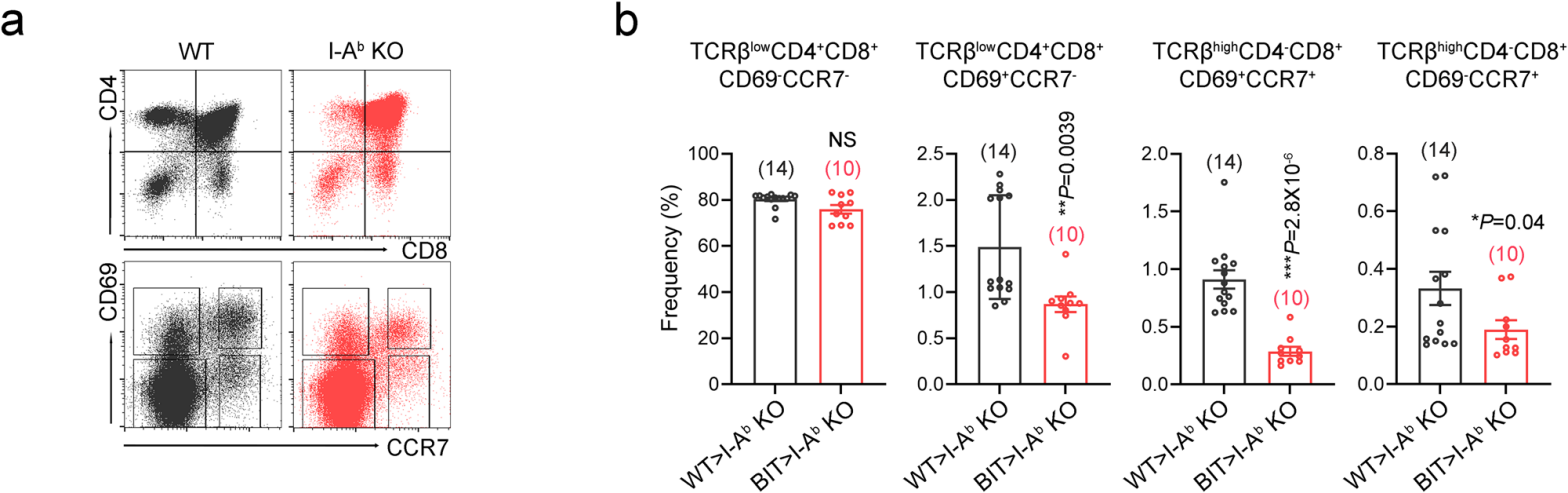
CD8⁺ T cell development in BIT mice is impaired as early as in cortical thymocytes. **a** Representative flow cytometric profiles of thymocytes isolated from WT (*n* = 14) and I-A^b^ KO (*n* = 10) mice. Data are representative of 3 independent experiments. **b** Thymocytes isolated from indicated bone marrow chimeric mice (WT > I-A^b^-KO, *n* = 14; BIT > I-A^b^-KO, *n* = 10) were analyzed by flow cytometry eight weeks after the reconstitution. The frequency of indicated cell populations in individual thymuses is plotted. Data are presented as means ± SEMs. *P < 0.05, ***P < 0.001, NS, not significant. Statistical analysis was performed using a two-tailed unpaired t-test.

Unlike WT mice, I-A^b^-KO mice were deficient in CD4-lineage thymocyte development (Fig. 5a). Similarly, like in WT > I-A^b^-KO bone marrow chimeras, BIT-derived thymocytes in BIT > I-A^b^-KO mice lacked the development of CD4^+^ CD8^-^ TCR^high^ thymocytes. However, in comparison with WT > I-A^b^-KO mice, BIT > I-A^b^-KO mice exhibited a moderate reduction in the generation of CD4^-^CD8^+^ TCR^high^ thymocytes, including CD4^-^CD8^+^ TCR^high^ CCR7^+^ medullary thymocytes (Fig. 5b). These results indicate that BIT hindrance of CD8^+^ T cell development was detectable in the absence of CD4-lineage T cell development. More importantly, CD4^+^ CD8^+^ TCR^low^ CD69^+^ CCR7^−^ thymocytes, which represented the cells that have been TCR-engaged for positive selection in the thymic cortex^24,25^, were already reduced in BIT > I-A^b^-KO mice in comparison with WT > I-A^b^-KO mice (Fig. 5b and Supplementary Fig. 4). These results indicate that thymoproteasome-expressing BIT hematopoietic cells hinder CD8-lineage T cell development in the thymus as early as the MHC-I-dependent positive selection stage of thymocytes within the thymic cortex. These results also indicate that BIT hindrance is neither initiated in the thymic medulla nor caused by the lack of proteasome switch between positively selecting cTECs and medullary APCs, including mTECs and hematopoietic APCs.

### No enhanced apoptosis during CD8^+^ T cell development in BIT mice

**Fig. 6:**
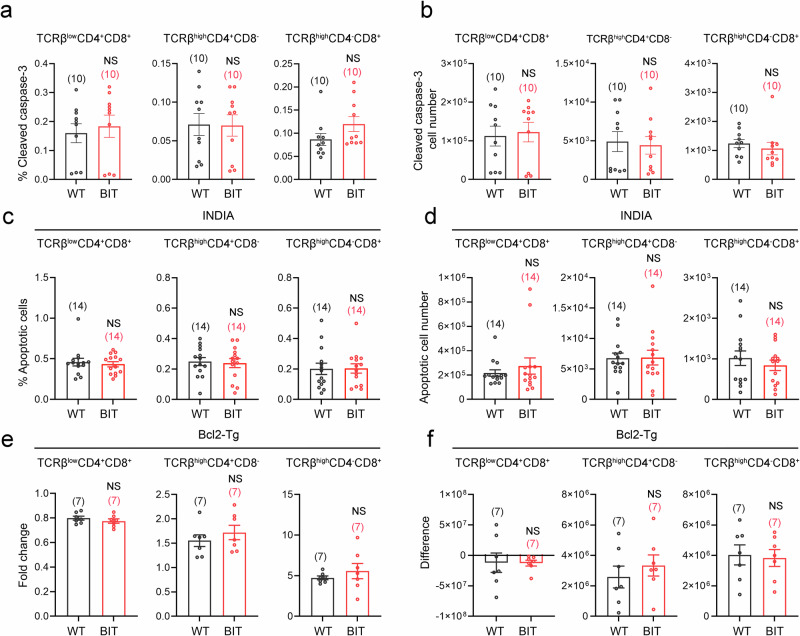
No increase in apoptosis was detected during CD8⁺ T cell development in BIT mice. Frequencies (**a**) and absolute numbers per mouse (**b**) of cleaved caspase-3 detected in indicated thymocyte populations from WT (*n* = 10) or BIT (*n* = 10) mice. Data are obtained from 3 independent experiments. Frequencies (**c**) and absolute numbers per mouse (**d**) of FRET-negative caspase 3-active cells in indicated thymocyte populations of INDIA mice (WT, *n* = 14; BIT, *n* = 14). Data are obtained from 8 independent experiments. Fold changes (**e**) and differences (**f**) in cell numbers of indicated thymocyte populations between Bcl2-Tg-positive and -negative mice (WT, *n* = 7; BIT, *n* = 7). Data are obtained from 7 independent experiments. Data are presented as means ± SEMs. NS, not significant. Statistical analysis was performed using a two-tailed unpaired t-test.

We further investigated whether BIT hindrance of CD8^+^ T cell development was accompanied by enhanced apoptosis of developing thymocytes, because impaired thymocyte development was often associated with enhanced negative selection of thymocytes, and because the corticomedullary proteasome switch hypotheses predicted enhanced negative selection of medullary thymocytes. To do so, an active form of caspase 3 was detected using a monoclonal antibody in various developmental stages of thymocytes isolated from WT and BIT mice (Supplementary Fig. 5a). We found that the frequency and number of caspase 3-active cells within developing thymocytes, including CD4^+^ CD8^+^ TCRβ^low^ cortical thymocytes and CD4^−^CD8^+^ TCRβ^high^ medullary thymocytes, were comparable and not significantly different between WT and BIT mice (Fig. 6a, b). Caspase 3 activity within developing thymocytes was also detected in Rosa26^INDIA^ knock-in mice wherein the loss of fluorescence resonance energy transfer (FRET) signals enabled the highly sensitive measurement of caspase 3 cleavage in cells^26,27^ (Supplementary Fig. 5b). We found that the frequency and the number of caspase 3-active cells detected from the loss of Rosa26^INDIA^ FRET were similar between WT and BIT mice throughout thymocyte development, from CD4^+^ CD8^+^ TCRβ^low^ immature thymocytes to CD4^−^CD8^+^ TCRβ^high^ mature thymocytes (Fig. 6c, d). Within CD4^+^ CD8^+^ TCRβ^low^ thymocytes, CD69^-^ thymocytes before TCR engagement and CD69^+^ TCR-engaged thymocytes exhibited equivalent frequency of caspase 3-active cells between WT and BIT mice (Supplementary Fig. 5c). Thus, no enhancement of caspase 3-dependent apoptosis was detected during CD8^+^ T cell development in the thymus of BIT mice, even by the use of highly sensitive Rosa26^INDIA^ FRET measurement.

Transgenic overexpression of antiapoptotic Bcl-2 prevents apoptosis-mediated negative selection in developing thymocytes^28,29^. We further examined the contribution of apoptosis-dependent negative selection to the reduced CD8^+^ T cell development in BIT mice, in the presence or absence of proximal Lck promoter-driven Bcl-2 overexpression^29^. We found that the Bcl-2 transgene affected CD8^+^ T cell development equivalently in WT and BIT mice (Fig. 6e, f). These results collectively demonstrate that there is no enhancement in apoptosis-mediated negative selection detected during hindered CD8^+^ T cell development in the thymus of BIT mice.

### Hindrance of CD8^+^ T cell development in BIT mice is dependent on β5t expressed in cTECs

**Fig. 7:**
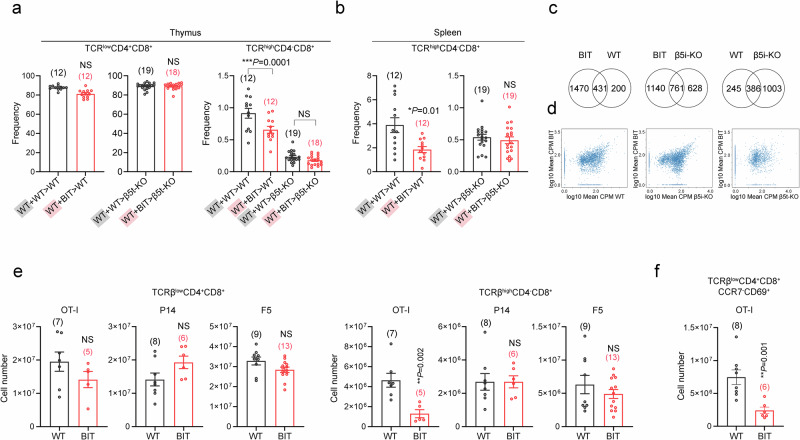
BIT hindrance in β5t-deficient thymus and in TCR-transgenic thymocytes. Indicated mixed bone marrow chimeric mice were analyzed eight weeks after the reconstitution. Thymocytes (**a**) and splenocytes (**b**) were analyzed by flow cytometry for CD4, CD8, and TCRβ surface expression. Graphs indicate frequencies of color-highlighted bone marrow-derived cells (grey or red) in indicated cell populations. Data are presented as means ± SEMs. Numbers in parentheses indicate sample numbers. **a** WT + WT > WT (*n* = 12), WT + BIT > WT (*n* = 12), WT + WT> β5t-KO (*n* = 19), and WT + BIT > β5t-KO (*n* = 18). **b** WT + WT > WT (*n* = 12), WT + BIT > WT (*n* = 12), WT + WT> β5t-KO (*n* = 19), and WT + BIT > β5t-KO (*n* = 19). Data are representative of 8 independent experiments. ***P < 0.001, NS, not significant. Statistical analysis was performed using an unpaired two-tailed t-test for comparisons between two groups and one-way ANOVA followed by Šidák’s multiple comparisons test for comparisons among multiple groups. **c** Venn diagrams illustrating unique and shared TCRα CDR3 sequences of CD8^+^ T cells, defined as those detected in at least three of four WT mice (631 sequences), five β5i-KO mice (1389 sequences), and four BIT mice (1901 sequences). **d** Scatter plots illustrating shared and unique TCRα CDR3 sequences of CD8^+^ T cells between BIT and WT mice, BIT and β5i-KO mice, and BIT and β5t-KO mice. Sequences expressed at ≥10 CPM in at least three biological replicates of at least one sample type were included. Scatter plots show normalized values. Numbers of mice analyzed were WT, *n* = 4; β5i-KO, *n* = 5; β5t-KO, *n* = 4; and BIT, *n* = 4. **e** Indicated thymocyte populations from TCR-transgenic Rag1-deficient mice were analyzed by flow cytometry for the surface expression of CD4, CD8, and TCRβ. Data are presented as means ± SEMs. Numbers in parentheses indicate the number of mice analyzed in each group. OT-I, WT (*n* = 7), BIT (*n* = 5); P14, WT (*n* = 8), BIT (*n* = 6); F5, WT (*n* = 9), BIT (*n* = 13). Data are obtained from 5–8 independent experiments. **P < 0.01, NS, not significant. Statistical analysis was performed using an unpaired two-tailed *t*-test. **f** Indicated thymocyte populations from OT-I TCR-transgenic Rag1-deficient mice (WT, *n* = 8; BIT, *n* = 6) were analyzed by flow cytometry for the surface expression of CD4, CD8, TCRβ, CD69, and CCR7. Data are obtained from 3 independent experiments. **P < 0.01. Statistical analysis was performed using an unpaired two-tailed t-test.

The results so far demonstrate that MHC-I-dependent antigen-presentation by ectopically thymoproteasome-expressing hematopoietic APCs hinders MHC-I-dependent development of immature thymocytes in the thymic cortex. MHC-I-dependent cortical development of thymocytes is profoundly but not completely dependent on the thymoproteasome endogenously expressed by cTECs^4,7,8,14^. We finally addressed whether the thymoproteasome endogenously expressed in cTECs might contribute to hematopoietic hindrance of CD8^+^ T cell development in BIT mice. In other words, we examined whether hematopoietic BIT hindrance might affect thymoproteasome-independent CD8^+^ T cell development, which was minor but detectable in β5t-deficient thymic microenvironment. To this end, we engineered mixed bone marrow chimeric mice in which WT hematopoietic stem cells would develop in the non-hematopoietic environment of irradiated β5t-deficient mice in the presence or absence of BIT mouse-derived hematopoietic stem cells ([WT + BIT] > β5t-KO vs. [WT + WT] > β5t-KO). In contrast to WT recipients in [WT + WT] > WT chimeric mice, β5t-deficient recipients lacked thymoproteasomes in the thymic microenvironment. This led to severe impairment in WT bone marrow stem cell-derived CD8^+^ T cell development in [WT + WT] > β5t-KO chimeras compared with WT recipients (Fig. 7a, b). On the other hand, WT bone marrow stem cell-derived CD8^+^ T cell development in [WT + BIT] > WT chimeras was moderately impaired by the presence of interfering BIT hematopoietic cells (Fig. 7a, b). More interestingly, WT bone marrow stem cell-derived CD8^+^ T cell development detected in the thymus and the spleen of [WT + BIT] > β5t-KO chimeric mice was not further impaired despite the presence of BIT hematopoietic cells (Fig. 7a, b). It was technically challenging to evaluate moderate BIT hindrance in the context of severely impaired CD8^+^ T cell development in a β5t-deficient thymic microenvironment. However, these results indicate that the loss of thymoproteasomes expressed in cTECs nullifies hematopoietic cell-mediated hindrance of CD8^+^ T cell development in BIT mice. These results also indicate that CD8^+^ T cell development, which is heavily dependent on endogenous thymoproteasome-expressing cTECs, cannot be compensated for by ectopically thymoproteasome-expressing hematopoietic cells. Thus, ectopically thymoproteasome-expressing hematopoietic cells specifically hinder endogenous thymoproteasome-expressing cTEC-dependent positive selection within the thymic cortex.

### TCR specificity affects susceptibility to BIT hindrance

In the thymic cortex, immature thymocytes are positively selected predominantly by cTECs rather than other cells, including hematopoietic cells such as DCs and neighboring thymocytes^30–32^. Our results demonstrate that thymic hindrance of CD8^+^ T cell development in BIT mice is due to the competition in the thymic cortex between thymoproteasome-expressing cTECs, which are competent to induce T-cell positive selection, and ectopically thymoproteasome-expressing hematopoietic cells, which are incapable of inducing T-cell positive selection (Supplementary Fig. 6). Deep sequencing analysis of TCR CDR3α regions demonstrated that TCR specificities of CD8^+^ T cells in BIT mice were as diverse as those in WT mice, β5i-KO mice, and β5t-KO mice (Supplementary Fig. 7a, b). Importantly, however, the TCR repertoire of CD8^+^ T cells shared in four BIT mice was unequal to, although in part overlapped with, that shared in WT, β5i-KO, or β5t-KO mice (Fig. 7c, d). More interestingly, we noted that β5t-dependent CDR3α sequences, such as those including CALSDRGTNAYKVIF and CALSDRTGNYKYVF, were reduced in CD8^+^ T cells similarly in BIT mice and β5t-KO mice, whereas other β5t-dependent sequences, such as those including CALLGYKLTF and CALSYQGGRALIF, were diminished in CD8^+^ T cells from β5t-KO mice but not BIT mice (Supplementary Fig. 7c). These results suggested that β5t-dependent positive selection was hindered partially in BIT mice.

The direct impact of BIT hindrance on thymoproteasome-dependent positive selection in the thymic cortex further suggested the possibility that the difference in TCR specificity might differently affect susceptibility to the hindrance by thymoproteasome-expressing BIT hematopoietic cells. To test this possibility, we examined BIT hindrance in three different TCR-transgenic RAG1-deficient strains expressing different TCR specificities. We found that CD8^+^ T cell development was highly susceptible to BIT hindrance in OT-I-TCR-transgenic mice, whereas CD8^+^ T cell development in F5-TCR-transgenic or P14-TCR-transgenic mice was resistant to BIT hindrance (Fig. 7e). These results indicate that the difference in TCR specificity affects the susceptibility of CD8^+^ T cell development to BIT-mediated hindrance.

It was previously shown that TCR signal intensity for cortical positive selection is higher in OT-I-TCR than P14-TCR or F5-TCR (OT-I > P14 > F5)^33^, and that the dependency on thymoproteasomes in cTECs is lower in OT-I-TCR than F5-TCR or P14-TCR (OT-I < F5 < P14)^7,34^. Thus, the susceptibility to BIT hindrance may proportionally correlate with TCR signal intensity and inversely with thymoproteasome dependency in cortical positive selection of CD8^+^ T cells. Together, these results support the notion that BIT hindrance reflects the interference of positive selection by endogenously thymoproteasome-expressing cTECs with ectopically thymoproteasome-expressing hematopoietic APCs.

We further found that BIT hindrance in OT-I-TCR-transgenic RAG1-deficient mice was detectable even at the CD4^+^ CD8^+^ TCR^low^ CD69^+^ CCR7^−^ cortical thymocyte stage (Fig. 7f), reinforcing the possibility that BIT hematopoietic cells hinder CD8^+^ T cell positive selection in the thymic cortex.

## Discussion

The present study describes a mouse model in which β5t-containing thymoproteasomes are ectopically expressed instead of β5i-containing immunoproteasomes. By utilizing these animals, we addressed the contribution of the β5t-dependent proteasome switch hypothesis. The proteasome switch hypothesis predicted that cTEC-specific expression of the thymoproteasome would lead to the difference in MHC-I-associated self-peptides expressed by positive-selection-inducing cTECs and negative-selection-inducing medullary APCs, including mTECs, so that positively selected thymocytes would include an optimal array of TCR specificities that could escape from subsequent negative selection in the thymic medulla^11,12,35,36^. It was therefore presumed that the ectopic expression of thymoproteasomes in the thymic medulla would cancel out the effect of differential proteasome expression, thereby enhancing medullary negative selection and compromising CD8^+^ T cell development. Contrary to the presumptive hypothesis, however, our results demonstrate that the ectopic expression of thymoproteasomes in mTECs did not impair thymoproteasome-dependent development of CD8^+^ T cells, indicating that the proteasome switch between cTECs and mTECs is dispensable for thymoproteasome-dependent development of CD8^+^ T cells.

Our results agree with the previously reported results showing that the genetically engineered proteasome switch between the immunoproteasomes expressed by cTECs and the constitutive proteasomes expressed by medullary APCs is not sufficient for the optimal development of CD8^+^ T cells^8^, indicating that the proteasome switch per se does not necessarily promote CD8^+^ T cell development. Nonetheless, the substrate-binding structures are much more different between the thymoproteasomes and the immunoproteasomes than those between the immunoproteasomes and the constitutive proteasomes^4,5,37^. MHC-I-associated peptides between immunoproteasome-expressing cTECs and constitutive proteasome-expressing medullary APCs in the previously engineered β5t^β5i^-knock-in β5i-deficient mice may not be as different and impactful as those between thymoproteasome-expressing cTECs and immunoproteasome-expressing medullary APCs in normal mice. On the contrary, the results presented in this study demonstrate that thymoproteasome-expressing cTECs fully optimize CD8^+^ T cell development even in the presence of thymoproteasome-expressing mTECs and in the absence of the β5t-dependent corticomedullary proteasome switch.

Our results further indicate that ectopically thymoproteasome-expressing hematopoietic cells hinder the positive selection of CD8^+^ T cells in the thymic cortex. This hindrance is dependent on MHC-I molecules expressed by hematopoietic cells and on thymoproteasomes expressed by cTECs. It is likely that the ectopically expressed thymoproteasomes promote the ectopic presentation of thymoproteasome-dependent MHC-I-associated self-peptides by hematopoietic cells, including DCs and neighboring thymocytes, localized in the thymic cortex. In normal mice, these thymoproteasome-dependent peptide-MHC-I complexes are limited to cTECs, which are efficient in inducing the positive selection of MHC-I-restricted CD8^+^ T cells. We propose that thymoproteasome-dependent peptide-MHC-I complexes ectopically expressed by cortical hematopoietic cells include peptide-MHC-I complexes that would compete with thymoproteasome-dependent peptide-MHC-I complexes endogenously expressed by cTECs, which are capable of engaging TCRs expressed by CD4^+^ CD8^+^ thymocytes. Consequently, ectopically thymoproteasome-expressing cortical hematopoietic cells would hinder thymoproteasome-dependent positive selection of CD8^+^ T cells induced by cTECs (Supplementary Fig. S6). We think it is conceivable that thymoproteasome-dependent alteration of MHC-I-associated peptides between cTECs and hematopoietic cells in the normal thymic cortex facilitates thymoproteasome-mediated positive selection of CD8^+^ T cells.

In contrast to endogenously thymoproteasome-expressing cTECs, our results showed that ectopically thymoproteasome-expressing hematopoietic cells did not support positive selection of cortical thymocytes. This may be due to the paucity of adhesion molecules in hematopoietic cells, unlike cTECs^38^. Adhesion molecules including VCAM-1 are abundant in cTECs, likely supporting proximal interactions between cTECs and CD4^+^ CD8^+^ thymocytes to allow TCR engagement by thymoproteasome-dependent self-peptide-MHC-I complexes^38,39^.

It is also interesting to speculate on the fate of cortical thymocytes that interact with ectopically thymoproteasome-expressing hematopoietic cells. These cortical thymocytes may be rapidly engulfed by phagocytes and eventually die in the thymic cortex, similar to unselected CD4^+^ CD8^+^ thymocytes that undergo “death-by-neglect”^40,41^, even though our apoptosis assays did not readily detect their cell death. In this regard, it is interesting to note that even when the positive selection in the light zone of germinal centers is inhibited, the increase in apoptosis of the light zone B cells is not detected while these B cells are destined to die^26^. Alternatively, thymocytes that engage with ectopically thymoproteasome-expressing hematopoietic cells may be diverted to an alternative lineage of thymocyte development, for example, toward the lineage of innate memory cells^42^.

It was previously reported that the systemic overexpression of β5t transgene impaired CD8^+^ T cell development in mice^43^, which partly seems to agree with our results. However, it was also shown that those systemically β5t-overexpressing transgenic mice suffered from reductions in body weight and lifespan, owing to the systemic interference of β5-containing constitutive proteasomes^44^. Thus, it was difficult to specifically discuss the mechanism of impaired CD8^+^ T cell development in those mice. In fact, our attempts to engineer the mouse genome to replace the β5-encoding *Psmb5* sequence with the β5t-encoding *Psmb11* sequence failed to produce live homozygous animals (unpublished results). It is likely that β5t is unable, or hardly able, to replace β5 and maintain the cells systemically in mice. In contrast, the replacement of β5i-encoding *Psmb8* with β5t-encoding *Psmb11* in BIT mice caused neither body weight nor lifespan reduction in the animals. We detected no reduction in the number of APCs, including DCs, B cells, and mTECs, even with the ectopic expression of thymoproteasomes in BIT mice (Supplementary Fig. 8a). Furthermore, BIT mice demonstrated no elevation of inflammatory lesions in various tissues (Supplementary Fig. 8b). Thus, we think it is plausible that β5t can replace β5i to assemble functional thymoproteasomes instead of immunoproteasomes, whereas β5t is unable, or hardly able, to replace β5 in the assembly and/or function of constitutive proteasomes.

Finally, our results indicated that the TCR repertoire of CD8^+^ T cells in BIT mice was as diverse as that in WT, β5i-KO, and β5t-KO mice. Transcriptomic and functional analyses also showed that CD8^+^ T cells generated in BIT mice appeared conventional and were not remarkably altered from WT CD8^+^ T cells (Supplementary Fig. 9). However, the results also showed that the repertoire of CD8^+^ T cells in BIT mice was unequal to, although partially overlapped with, that in WT, β5i-KO, and β5t-KO mice. We previously reported that the TCR repertoire of CD8^+^ T cells was altered in β5t-deficient mice in comparison with that in WT mice^14^. Interestingly, our results further suggested that β5t-dependent positive selection was partially hindered in BIT mice. However, it is important to note that BIT mice exhibited no signs of autoimmune inflammatory lesions in a variety of organs (Supplementary Fig. 8b), like β5t-deficient mice^7^. This is likely due to unimpaired machinery for T-cell self-tolerance, including the deletion of self-reactive T cells and the generation of regulatory T cells^1,2,45,46^, in the thymic medulla and the periphery in β5t-deficient mice and BIT mice. Indeed, we previously showed that the lack of β5t did not aggravate autoimmune disease in mice lacking RelB-dependent mTECs^14^.

In conclusion, the present results demonstrate that the ectopic expression of thymoproteasomes in mTECs does not compromise the thymoproteasome-dependent positive selection of CD8^+^ T cells, arguing against the proteasome switch hypothesis for the mechanism of thymoproteasome-dependent CD8^+^ T cell development. The thymoproteasome controls CD8^+^ T-cell development without a proteasome switch between the positive selection-inducing thymic cortex and the negative selection-inducing thymic medulla. Our results further point to a novel mechanism of thymoproteasome-dependent CD8^+^ T cell development, in which the alteration of MHC-I-associated peptides between cTECs and hematopoietic cells in the thymic cortex facilitates thymoproteasome-mediated positive selection of CD8^+^ T cells. These findings have significant implications for advancing our understanding of the mechanisms underlying T cell positive selection by highlighting the contribution of previously unknown mechanisms in securing the unique capability of cTECs, rather than other cortical APCs, to induce positive selection in the thymic cortex.

## Methods

### Mice

C57BL/6 (B6) mice and B6-CD45.1 (B6.SJL-*Ptprc*^*a*^*Pepc*^*b*^) mice were obtained from The Jackson Laboratory. B6 mice were also obtained from Charles River Laboratories. β5t-deficient mice^4,17^, β5i-deficient mice^19^, TCRα-deficient mice^47^, Rag1-deficient mice^48^, β2m-deficient mice^49^, I-A^b^-deficient mice^50^, Rosa26^INDIA^-knockin mice^26^, *pLck*-Bcl2-transgenic mice^28^ were previously described. All mice were backcrossed to the B6 background and maintained in our animal facility. Mice were maintained under specific pathogen-free conditions on 12-h light-dark cycle at 20–26 °C with 30–70% humidity in accordance with US National Institute of Health guidelines. All mouse experiments were performed under consent by the NCI at Frederick Animal Care and Use Committee of the National Cancer Institute (ASP21-431, ASP21-432, and EIB-076).

### Engineering of BIT mice

Guide RNAs targeting around the first and last protein-coding exons of *Psmb8* were designed using sgRNA Scorer 2.0^51^ and tested for in vitro cutting activity in P19 cells^52^ with candidates #718, #727, and #759 subsequently selected for mouse editing experiments (Supplementary Table 1). To generate the mice in which only the first protein exon of *Psmb8* was replaced by *Psmb11* (BIT#1, Supplementary Fig. 1), a plasmid donor was made containing ~500 bp of homology on the 5’ and 3’ ends of the first protein coding exon, along with *Psmb11* cDNA sequence. Briefly, this was done by isothermal assembly^53^ using a synthesized DNA fragment (Twist Biosciences) and the pGMC00018 (Addgene #195320) plasmid. Upon sequence verification of the cloned plasmid using Sanger sequencing, single-stranded DNA (pAG0016-ssDNA) was generated using the Guide-it Long ssDNA Production System (Takara). Synthetically modified versions of candidates #718 and #759, obtained from Synthego, were then complexed with recombinant Cas9 protein and ssDNA donor and microinjected into B6 mouse embryos using methodology previously described^54^. Similarly, for the generation of BIT#2 mice (Supplementary Fig. 1), modified versions of guide RNAs #718 and #727 were complexed with recombinant Cas9 protein and an ssDNA donor (pAG0017-ssDNA) containing ~500 bp of homology to sequence 5’ of the first protein coding exon and 3’ of the last protein coding exon along with *Psmb11* cDNA and microinjected into B6 mouse embryos as described above.

### Bone marrow chimeric mice

Bone marrow chimeric mice were generated by transplanting lethally irradiated mice (9.5 Gy) with 2.5 × 10^6^ T cell-depleted bone marrow cells. For mixed chimeras, recipients received a 1:1 mixture of bone marrow cells from two donor genotypes, totaling 2.5 × 10^6^ cells per recipient. Cells from two kinds of bone marrow cells and from recipient mice were distinguished by the preparation of chimeric mice using the combination of B6, B6-CD45.1, and their F1 mice and the identification of CD45.1^+^CD45.2^−^, CD45.1^−^CD45.2^+^, and CD45.1^+^CD45.2^+^ cells by using monoclonal antibodies specific for CD45.1 and CD45.2. Mice were maintained on antibiotic-supplemented water for 2 weeks post-transplantation. Thymocytes and splenocytes were analyzed 6–8 weeks after the reconstitution.

### Flow cytometry and cell sorting

For the analysis of thymocytes and splenocytes, cells were stained with antibodies specific for anti-mouse CD8α (clone 53–6.7, BioLegend, cat#100725, 1:100), CD4 (clone RM4-5, BioLegend, cat#100512, 1:100), TCRβ (clone H57-597, BioLegend, cat#109218, 1:50), CD69 (clone H1.2F3, BioLegend, cat#104507, 1:100), and CCR7 (clone 4B12, R&D Systems, FAB3477R-100UG, 1:100). Staining for CCR7 was performed at 37 °C for 30 min. For the analysis of DCs and B cells, cells were stained with antibodies specific for anti-mouse CD11c (clone N418, eBioscience, cat#47-0114-B2, 1:100) and CD19 (clone 1D3, eBioscience, cat#50-0193-82, 1:100), respectively. For the detection of MHC class I molecules, cells were stained with antibodies specific for H-2K^b^ (clone AF6-88.5, BioLegend, cat#116512, 1:100) and H-2D^b^ (clone 28-14-8, eBioscience, cat#12-5999-82, 1:100).

For the analysis of thymic epithelial cells (TECs), thymus tissues were minced and digested with 1 unit/ml Liberase TM (Roche, cat#5401119001) in the presence of 0.01% DNase I (Roche, cat#1010415900). Cells were stained with antibodies specific for anti-mouse CD45 (clone 30-F11, BioLegend, cat#103134, 1:40), EpCAM (clone G8.8, BioLegend, cat#118216, 1:200), and Ly51 (clone 6C3, BioLegend, cat#108312, 1:200), and for reactivity with UEA-1 (Vector Laboratories, cat#DL-1067, 1:200)^55^.

For the detection of intracellular β5t and cleaved caspase-3, cells were fixed and permeabilized using Foxp3/Transcription Factor Staining Buffer Set (eBioscience, cat#00-5523-00) in accordance with the manufacturer’s protocol. Cells were stained with monoclonal antibody specific for β5t (CPTC-PSMB11(mouse)−1, Antibody Characterization Program of the National Cancer Institute, https://antibodies.cancer.gov/browse) or cleaved caspase-3 (clone C92-605.rMAb, BD Pharmigen, cat#570185, 1:100).

For the detection of transcription factors in T cells, lymph node cells were surface-stained for TCRβ, CD4, and CD8, fixed and permeabilized with using Foxp3/Transcription Factor Staining Buffer Set (eBioscience, cat#00-5523-00) and stained with fluorescence-conjugated ThPOK (clone T43-94, BD Bioscience, cat#565500, 1:50), and Runx3 (clone R3-5G4, BD Bioscience, cat#564814, 1:25).

Flow cytometric analysis was performed on FACS LSR II or FACS Fortessa (BD Biosciences). Cell sorting was performed on FACSAria SORP (BD Biosciences). Prior to the sorting of TECs, CD45-negative cells were enriched using Mouse CD45 MicroBeads (Miltenyi Biotec, cat#130-052-301). Dead cells were excluded by forward light-scatter gating and staining with propidium iodide for freshly prepared cells or Fixable Ghost Dye (Cytek Biosciences) for fixed cells. Data were analyzed by using FlowJo FACS Analysis software (BD Biosciences). Flow cytometric gating strategies are shown in Supplementary Figure 10. Additional information for antibodies used in this study is listed in Supplementary Table 2.

### Fluorescence resonance energy transfer detection

Fluorescence resonance energy transfer (FRET) signal in cells from Rosa26^INDIA^-knockin mice was measured using excitation of mNeonGreen (donor fluorophore) and emission of both mNeonGreen and mRuby2 (acceptor fluorophore). FRET^+^ alive and FRET^−^ early apoptotic cells were defined based on the ratio of mNeonGreen to mRuby2 fluorescence among DAPI^negative^ cells and analyzed by flow cytometry, as previously described^26,27^.

### Immunoblot analysis

Cell lysates in RIPA lysis buffer (Thermo Fiser, cat#89900) supplemented with protease inhibitors (Cell Signaling, cat#5872) were clarified by centrifugation at 16,000 × *g* at 4 °C for 20 min. Protein concentration was measured with a BCA protein assay kit (Pierce). Total protein (100 μg) was denatured in SDS protein gel loading solution (Quality Biological) supplemented with 10 mM DTT at 95 °C for 10 min. Equal amounts of total proteins were electrophoresed in 12% NuPAGE Tris-glycine gels (Thermo Fisher) at 150 V for 2 h. The gels were transferred to positively charged PVDF membranes at 20 V for 7 min. The membranes were blocked with 5% (w/v) nonfat milk in TBST (25 mM Tris, 150 mM NaCl, 0.05% Tween 20, pH 7.2) and probed with either anti-β5t antibody (polyclonal rabbit antiserum, 1:1000)^4,56^ or anti-β5i antibody (rabbit D1K7X, Cell Signaling, cat#13635S, 1:1000) at 4 °C overnight, followed by washing with TBST and incubation with horseradish peroxidase-conjugated anti-rabbit IgG antibody (1:2500) at room temperature for 1 h. Membranes were also probed with horseradish peroxidase-conjugated anti-β-actin antibody (clone 2A3, Santa Cruz, cat#sc-517582HRP, 1:1000). Horseradish peroxidase activity was visualized with a chemiluminescent substrate (Thermo Fisher).

### Quantitative RT-PCR analysis

Total cellular RNAs extracted from TECs, dendritic cells, and B cells sorted from the thymus were reverse-transcribed (RT) with PrimeScript RT Master Mix (Takara). Quantitative real-time PCR was performed using SYBR Premix Ex Taq (Takara) and QuantStudio 6 Flex Real-time PCR System (Applied Biosystems). Amplified products were confirmed to be single bands in gel electrophoresis. Gene expression values were normalized to those of the *Gapdh* housekeeping gene.

### TCR sequencing analysis

Deep sequencing analysis of the TCRα CDR3 region was performed as previously described^14,57^. Briefly, 10⁶ CD8⁺ T cells were sorted from lymph nodes and spleens of WT, β5i-KO, β5t-KO and BIT mice (9–12 weeks old, male, *n* = 4–5 individual mice per group, >98% purity). RNA extraction and library preparation followed the procedures described previously^14^. TCR sequences were analyzed as previously reported, based on the predicted amino acid sequence of the CDR3 region. Highly confident and recurrently expressed CDR3 clones were selected for further analysis. Reads were normalized to counts per million (CPM), and only CDR3 clones present at ≥10 CPM in at least three biological replicates of at least one sample type were included^57^. Analysis was performed using MiXCR, Venny (v2.1), Python (v3.11.5) with packages NumPy, Pandas and matplotlib, and R (v4.3.1) using the EdgeR package. The data have been deposited in the Gene Expression Omnibus (GEO). The GEO accession ID is GSE322772.

### Immunofluorescence analysis of tissue sections

Thymus and spleen tissues were fixed with 4% paraformaldehyde and sliced into 10-μm-thick sections. The sections were stained with antibodies specific for Aire (clone 5H12, Invitrogen, cat#0-5934-82, 1:100), CD11c (clone N418, Biolegend, cat#117312, 1:100), CD3 (clone 17A2, eBioscience, cat#14-0032-82, 1:100), and B220 (clone RA3-6B2, BD Biosciences, cat#553090, 1:100). Images were obtained by using Nikon Eclipse Ti2 microscope (Nikon) and CSU-W1 spinning disk scanner (Yokogawa) with Hamamatsu Orca Flash 4.0 camera and analyzed with NIS-Elements software (Nikon) and ImageJ software (National Institutes of Health).

### Histopathological analysis

Tissues were fixed with 10% phosphate-buffered formalin (pH 7.2, Sigma). Paraffin sections were stained with hematoxylin and eosin (Histoserve). Images were analyzed by Zeiss ZEN (Zeiss). Pathological score was determined as follows: a score of 0 indicates no inflammation; 1 indicates slight inflammation with one to five foci, where each focus is composed of more than 20 mononuclear cells; 2 indicates moderate inflammation with more than five such foci but without significant parenchymal damage; and 3 indicates severe inflammation resulting from the degeneration of parenchymal tissue with lymphocyte infiltration^58^.

### In vivo T cell proliferation analysis

T cells (CD45.1^−^CD45.2^+^) purified from lymph nodes using a Pan T Cell Isolation Kit II (Miltenyi Biotec) were labeled with 0.5 μM Cell Trace Violet (Invitrogen) and injected intravenously into host B6 (CD45.1^+^CD45.2^−^) mice that were exposed to 6 Gy sublethal irradiation the previous day. The mice were analyzed six days after injection.

### RNA sequencing analysis of T cells

Total RNA from CD4^-^CD8^+^ T cells sorted from lymph nodes was extracted using an RNeasy Plus Mini Kit (Qiagen, cat#74034). RNA quality was assessed using a Bioanalyzer (Agilent). The library was prepared using a Stranded Total RNA Prep Ligation with Ribo-Zero Plus for sequencing (Illumina). Paired-end sequencing (length: 100 base pairs) was performed on a NextSeq 2000 (Illumina). Sequencing reads were trimmed using Cutadapt and aligned to the mouse reference genome (GRCm39) with a STAR aligner 85, and raw count data were generated using RSEM based on Gencode M38. For differential gene expression analysis, raw counts were normalized by quantile normalization, and statistical analysis was performed using the limma-voom algorithm. The data have been deposited in the Gene Expression Omnibus (GEO). The GEO accession ID is GSE319325.

### Statistical analysis

Statistical analysis was performed using GraphPad Prism 10 (GraphPad Software). For comparisons between two groups, an unpaired two-tailed Student’s *t* test was used. For experiments involving three or more groups, one-way ANOVA followed by Dunnett’s multiple comparisons test (for comparisons with WT) or Šídák’s multiple comparisons test (for multiple pairwise comparisons) was applied. For datasets with excessive zero values, non-parametric tests (Kruskal–Wallis) followed by Dunn’s multiple comparisons test were applied. *P* values of 0.05 or lower were considered statistically significant. All values are presented as means ± standard error of the means. Sample sizes (*n*) are indicated in the figures.

### Reporting summary

Further information on research design is available in the Nature Portfolio Reporting Summary linked to this article.

## Supplementary information

Supplementary Information

Reporting Summary

Transparent Peer Review File

## Source data

Source Data

## Data Availability

RNA-sequencing datasets are available in the Gene Expression Omnibus (GEO) with the accession IDs GSE319325 and GSE322772. Reagents are listed in Supplementary Table 2. The raw numbers for charts and graphs, as well as uncropped blot scans, are available in the Source Data file. All data are available from the corresponding author upon reasonable request. Source data are provided with this paper.
