# Supplementary material for: Proteasome alteration between epithelial and hematopoietic cells facilitates positive selection of CD8 T cells
Source: Nat Commun. 2026 Apr 27;17:5780. doi: 10.1038/s41467-026-72411-x (PMC13324746; doi:10.1038/s41467-026-72411-x)
Supplement: Supplementary file 1 — Supplementary Information [file 41467_2026_72411_MOESM1_ESM.pdf]

Supplementary Information

Title:
Proteasome alteration between epithelial and hematopoietic cells facilitates positive selection of CD8 T cells

Authors:
Mami Matsuda-Lennikov, Jamie-Jean De La Torre, Todd Snow, Jacqueline Battaile, Felix Kalle-Youngoue, Alison Jacques, Aya Ushio, Akihide Shimizu, Miho Shinzawa, Christian T. Mayer, Parirokh Awasthi, Raj Chari, Izumi Ohigashi, Shigeo Murata, Yousuke Takahama

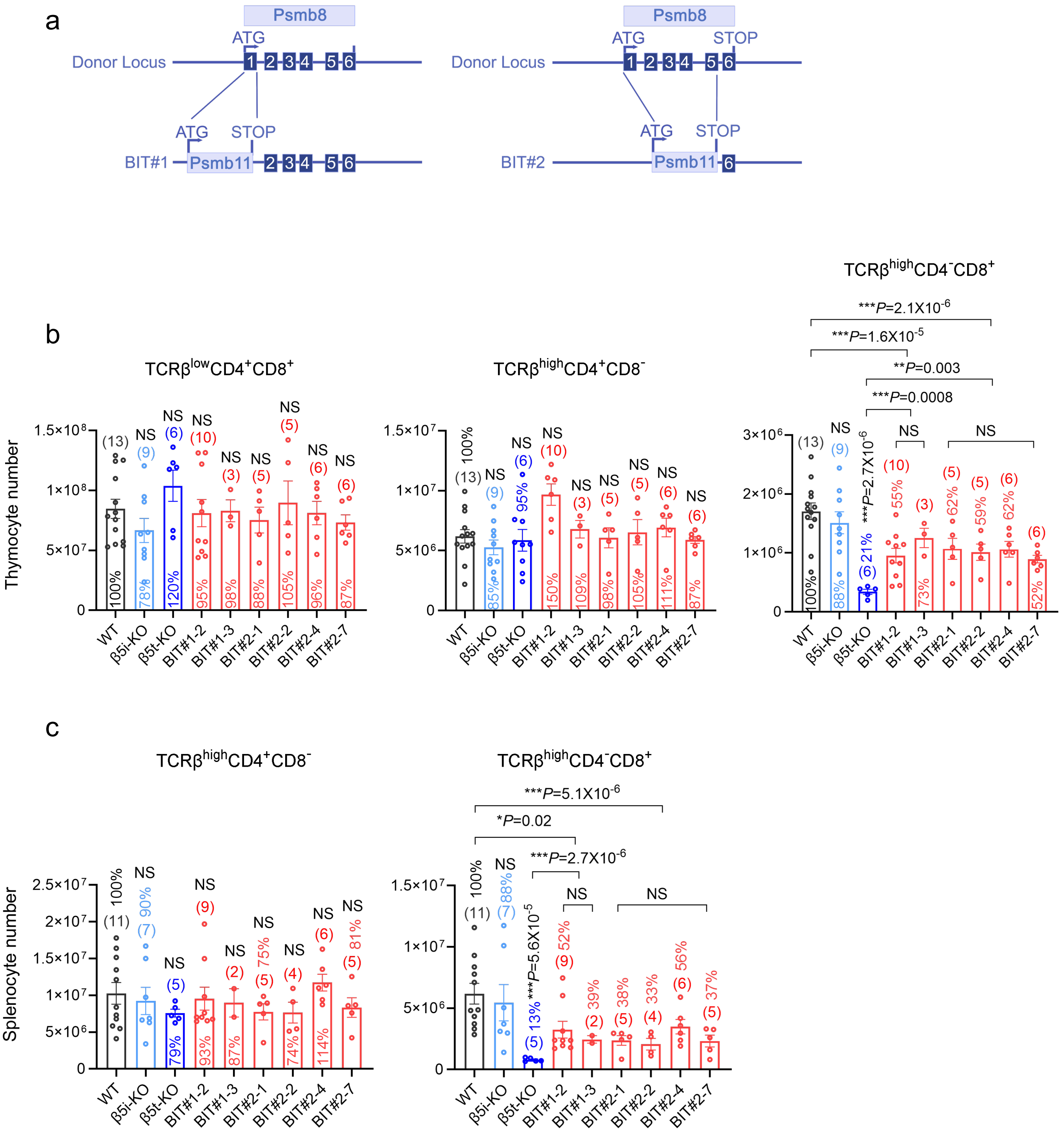

Supplementary Figure 1. Two different genomic modifications and six independent lines of BIT mice.
a. Scheme for engineering β5i^{β5t} (BIT) alleles. β5t-encoding *Psmb11* sequence was inserted either within exons 1 (left, BIT#1 as in **Fig. 1a**) or between exons 1 and 5 (right, BIT#2) of β5i-encoding *Psmb8* gene in the mouse genome.
b. and **c.** Absolute numbers (per mouse) of indicated populations of thymocytes (**b**) and splenocytes (**c**) from indicated mice at 4 to 6 weeks old. Bar graphs represent means ± standard errors. Dots represent the data from individual mice. Numbers in parentheses indicate the numbers of independent mice examined per group. **b.** WT, n=13; β5i-KO, n=9; β5t-KO, n=6; BIT#1-2, n=10; BIT#1-3, n=3; BIT#2-1, n=5; BIT#2-2, n=5; BIT#2-4, n=6; BIT#2-7, n=6. **c.** WT, n=11; β5i-KO, n=7; β5t-KO, n=5; BIT#1-2, n=9; BIT#1-3, n=2; BIT#2-1, n=5; BIT#2-2, n=4; BIT#2-4, n=6; BIT#2-7, n=5. Data from 2-10 independent experiments are shown. Statistical analysis was performed using one-way ANOVA followed by Dunnett's multiple comparisons test. *P < 0.05; **P < 0.01; ***P < 0.001; NS, not significant.

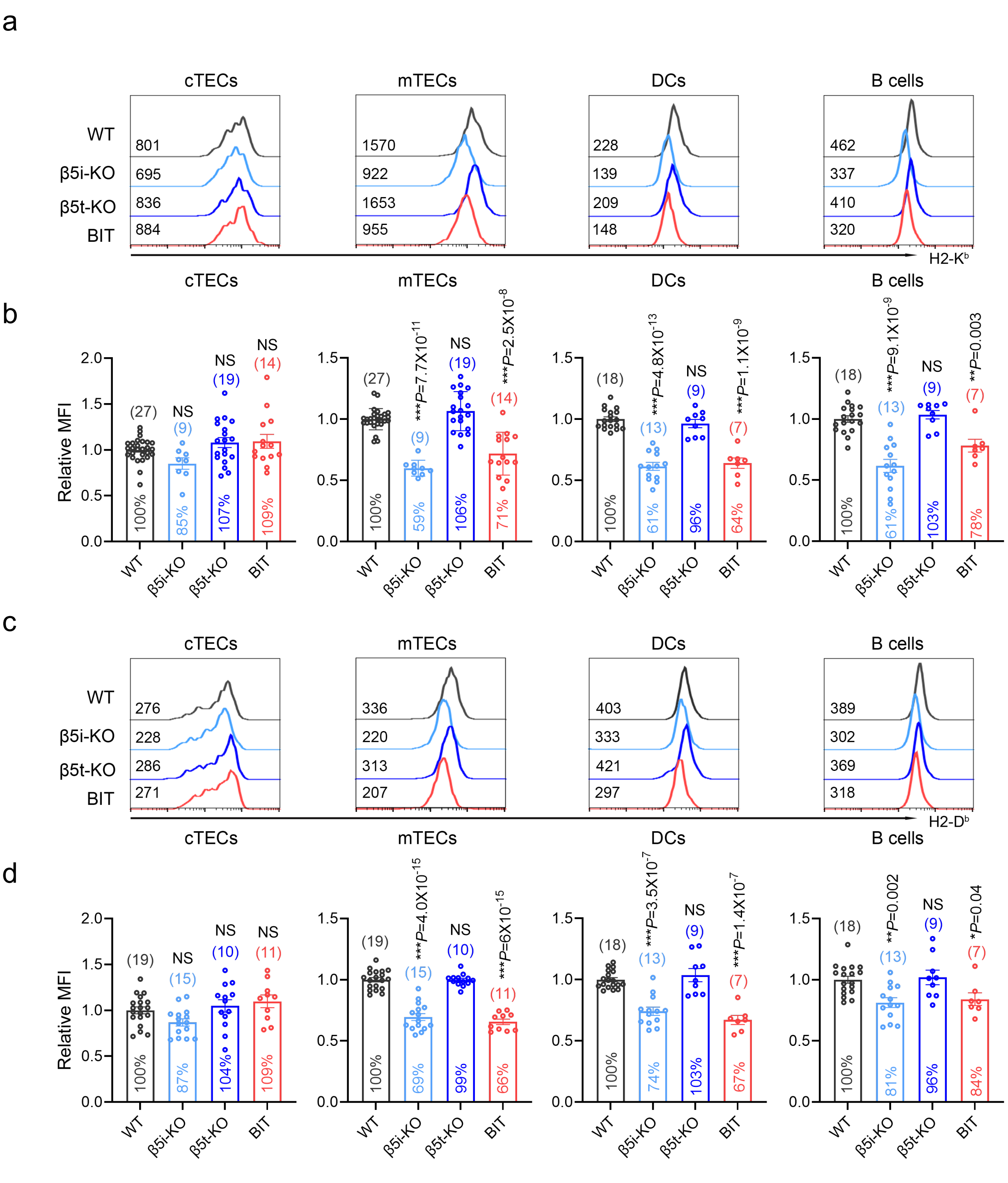

Supplementary Figure 2. MHC-I expression by thymic APCs in BIT mice.

a. and **b.** Flow cytometric analysis of surface MHC-I expression (H2-K^b) in indicated cells from the thymus of indicated mice.

c. and **d.** Flow cytometric analysis of surface MHC-I expression (H2-D^b) in indicated cells from the thymus of indicated mice.

Shown are representative histograms and individual mean fluorescence intensities (MFIs) (**a, c**) as well as means ± standard errors (sample numbers) of relative MFIs (**b, d**).

b. and **d.** Dots represent the data from individual mice. Numbers in parentheses indicate the numbers of independent mice examined per group. **b.** cTECs and mTECs; WT, n=27; β5i-KO, n=9; β5t-KO, n=19; BIT, n=14. DCs and B cells; WT, n=18; β5i-KO, n=13; β5t-KO, n=9; BIT, n=7. **d.** cTECs and mTECs; WT, n=19; β5i-KO, n=15; β5t-KO, n=10; BIT, n=11. DCs and B cells; WT, n=18; β5i-KO, n=13; β5t-KO, n=9; BIT, n=7. Data from 9-15 independent experiments are shown. Statistical analysis was performed using one-way ANOVA followed by Dunnett’s multiple comparisons test. *P < 0.05; **P < 0.01; ***P < 0.001; NS, not significant.

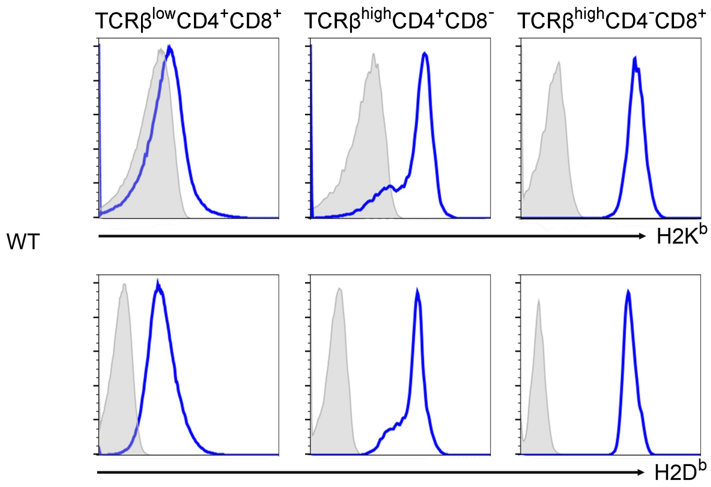

Supplementary Figure 3. MHC-I expression by developing thymocytes.
Flow cytometric analysis of surface MHC-I expression in indicated cell populations from the thymus of indicated mice. Gray histograms represent unstained controls. Data are representative of three independent experiments.

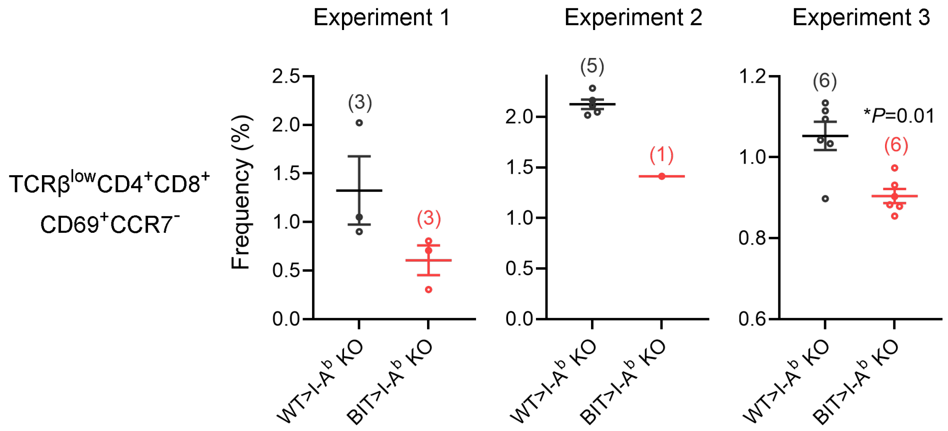

Supplementary Figure 4. CD8⁺ T cell development in BIT mice is impaired as early as in cortical thymocytes.

Thymocytes isolated from indicated bone marrow chimeric mice were analyzed by flow cytometry eight weeks after reconstitution. Shown is the frequency (means ± standard errors) of indicated cell populations in individual thymuses. Data from three individual bone marrow chimera preparations are plotted separately. Dots represent the data from individual mice. Experiment 1 (WT, n=3; BIT, n=3), experiment 2 (WT, n=5; BIT, n=1), and experiment 3 (WT, n=6; BIT, n=6). Numbers in parentheses indicate the numbers of independent mice examined per group. Data from 3 independent experiments are shown. Statistical analysis was performed using an unpaired two-tailed t-test. *P < 0.05.

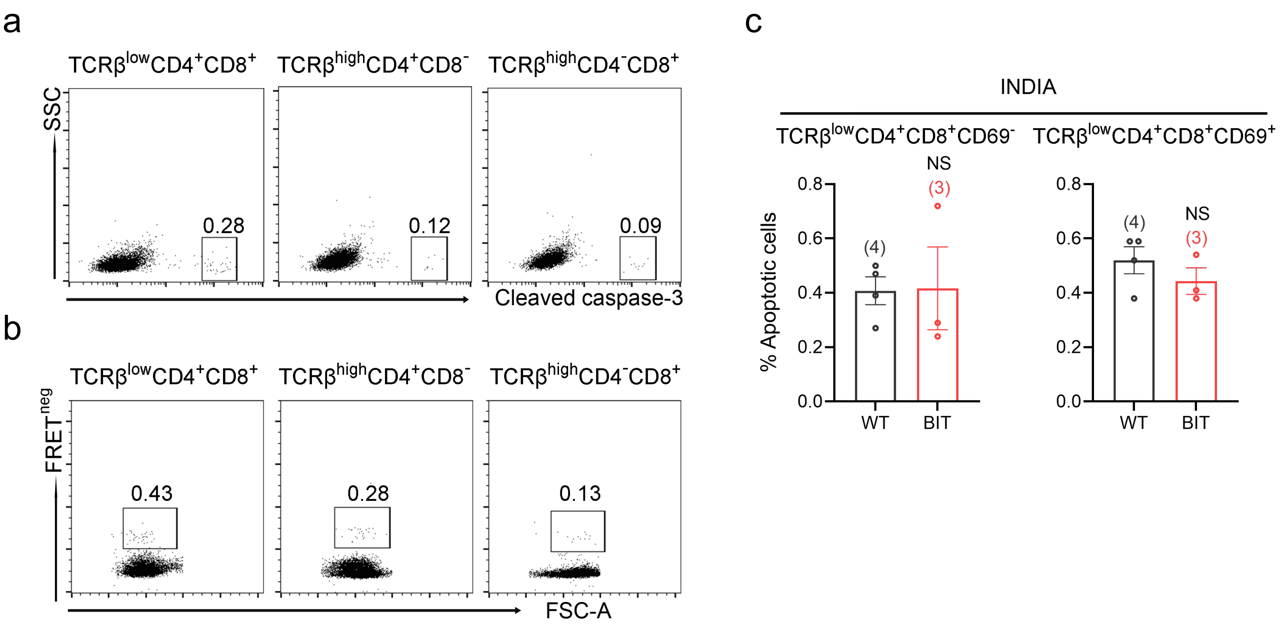

Supplementary Figure 5. Detection of apoptotic thymocytes in BIT mice.

a. Representative flow cytometric profiles for the detection of cleaved caspase-3 in thymocytes.

b. Representative flow cytometric profiles for the detection of FRET-negative caspase 3-active cells in INDIA thymocytes. Numbers above boxes indicate percentage of cells in indicated areas.

c. Frequency of FRET-negative caspase 3-active cells in indicated thymocyte populations of INDIA mice. Dots represent the data from individual mice. Numbers in parentheses indicate the numbers of independent mice examined per group (WT, n=4; BIT, n=3). Data from three independent experiments are shown. Statistical analysis was performed using an unpaired two-tailed t-test. NS, not significant. CCR7 was excluded from this analysis because CCR7 staining requires incubation at 37 °C, affecting INDIA FRET profiles.

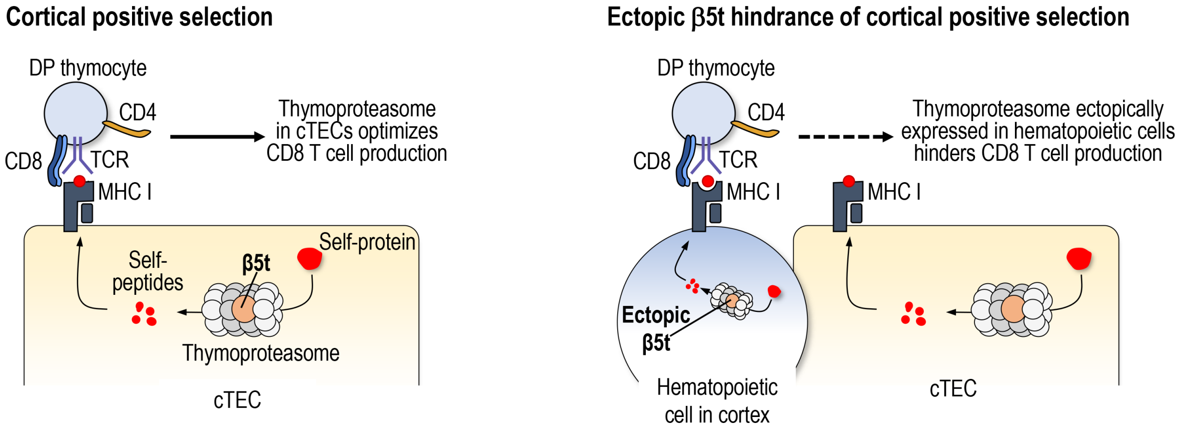

Supplementary Figure 6. Scheme for BIT hindrance.
In normal thymic cortex (left), thymoproteasome-expressing cTECs present peptide-MHC-I complexes that efficiently mediate the positive selection of $CD8^{+}$ T cells. In BIT mice (right), ectopic expression of thymoproteasomes in hematopoietic cells results in the presentation in the thymic cortex of thymoproteasome-dependent peptide-MHC-I complexes, which interfere with the positive selection by competing with cTEC-mediated engagement of TCRs expressed by $CD4^{+}CD8^{+}$ thymocytes.

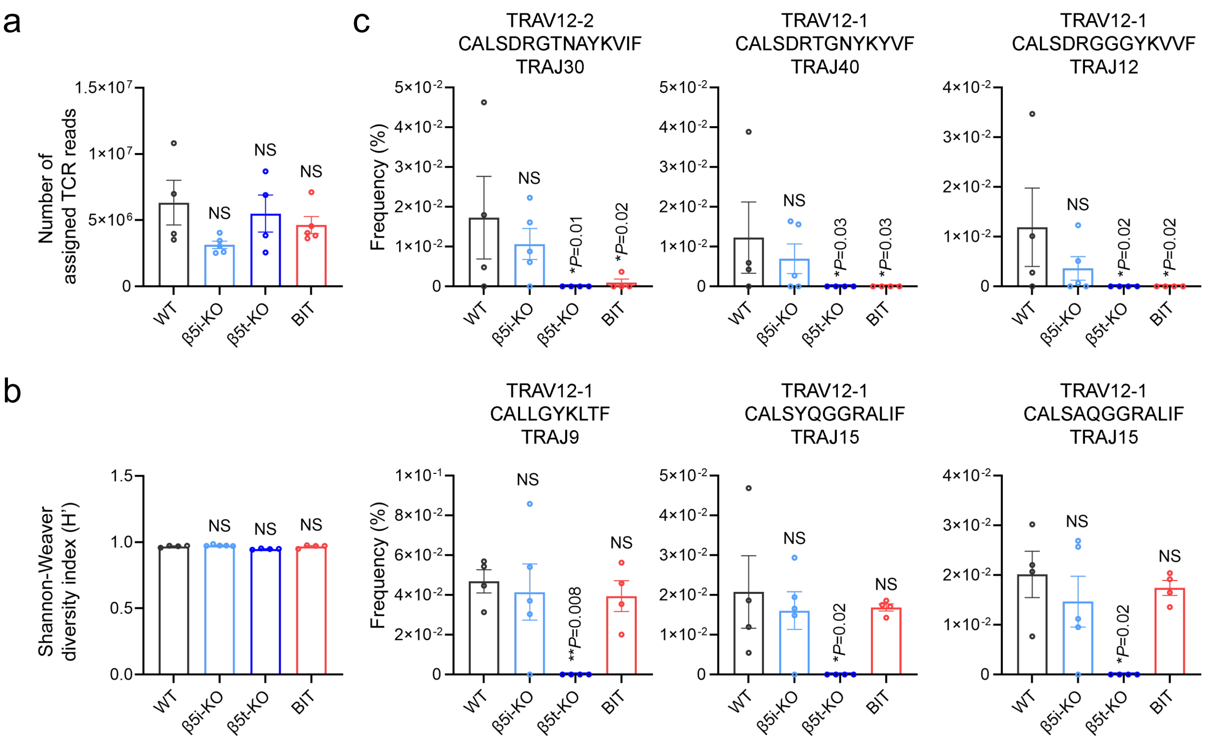

Supplementary Figure 7. TCR sequencing analysis of BIT mice.

a. Numbers (means and SEMs, n=4-5 in five independent measurements) of TCRα CDR3 sequences assigned to in-frame V-J rearranged TCRa in 10^6 $CD8^+$ T cells from four to five individual mice.

b. Shannon-Weaver diversity indexes of TCRa CDR3 diversity in $CD8^+$ T cells from indicated mice.

c. Representative β5t-dependent TCRα CDR3 sequences in $CD8^+$ T cells that are susceptible (top) and resistant (bottom) to BIT hindrance.

Dots represent the data from individual mice (WT, n = 4; β5i KO, n = 5; β5t KO, n = 4; BIT, n = 4). Each dot represents an individual mouse. Statistical analysis was performed using one-way ANOVA followed by Dunnett's multiple comparisons test (**a**, **b**) and Kruskal-Wallis test followed by Dunn's multiple comparisons test (**c**). *P < 0.05; **P < 0.01; NS, not significant.

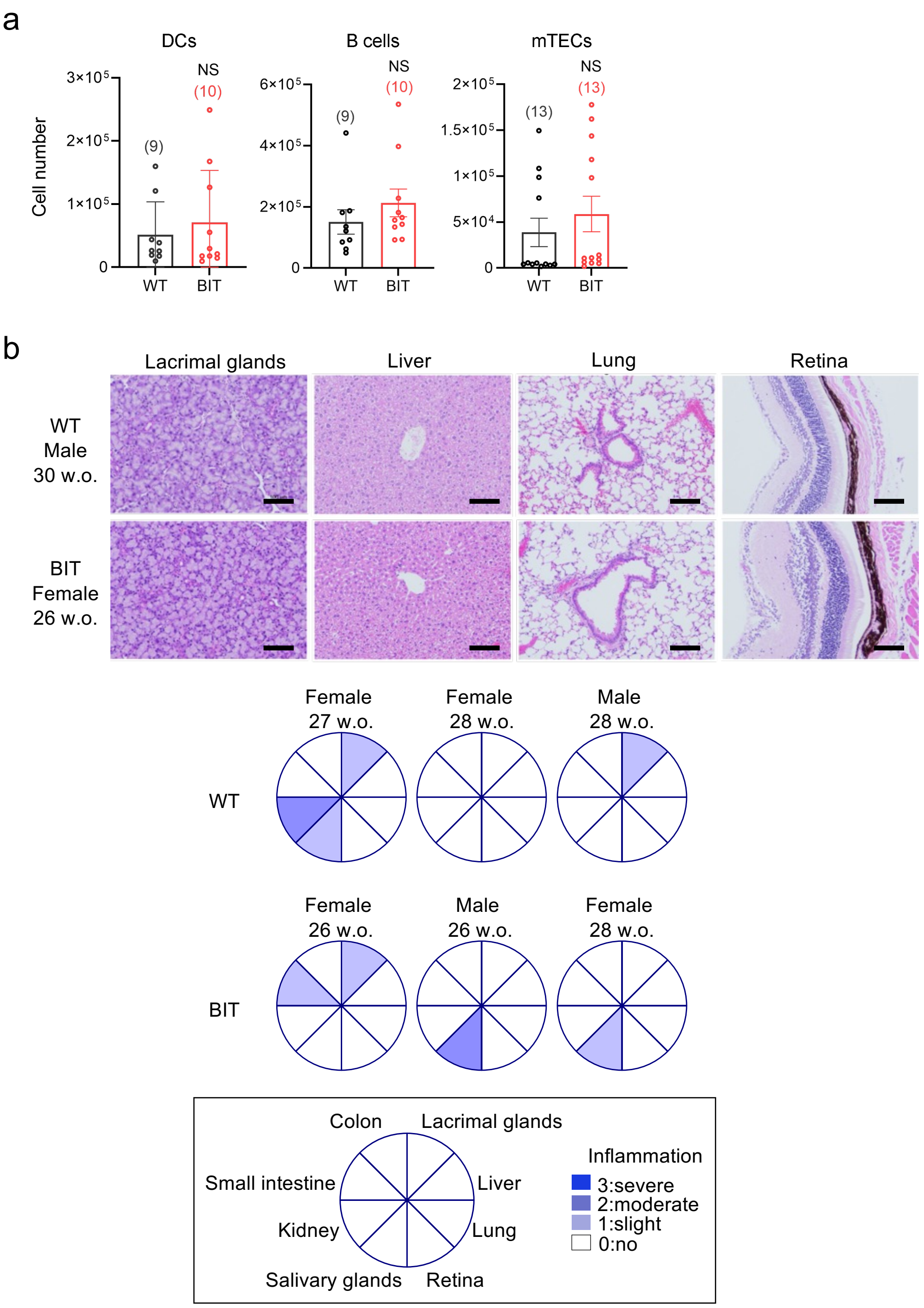

Supplementary Figure 8. Histopathological analysis of BIT mice.
a. Absolute numbers (per mouse) of indicated cell populations in the thymus from indicated mice at 2 to 8 weeks old. Bar graphs represent means ± standard errors. Dots represent the data from individual mice. Data are representative of three independent experiments. Numbers in parentheses indicate the numbers of independent mice examined per group. DCs and B cells, WT, n=9; BIT, n=10. mTECs, WT, n=13; BIT, n=13. Statistical analysis was performed Statistical analysis was performed using an unpaired two-tailed t-test. NS, not significant.
b. Paraffin sections of formalin-fixed tissues were stained with hematoxylin and eosin. Data are representative of three independent experiments. Shown are representative images (top) and pathological scores (bottom) of indicated tissues in indicated mice. Scale bars, 100μm.

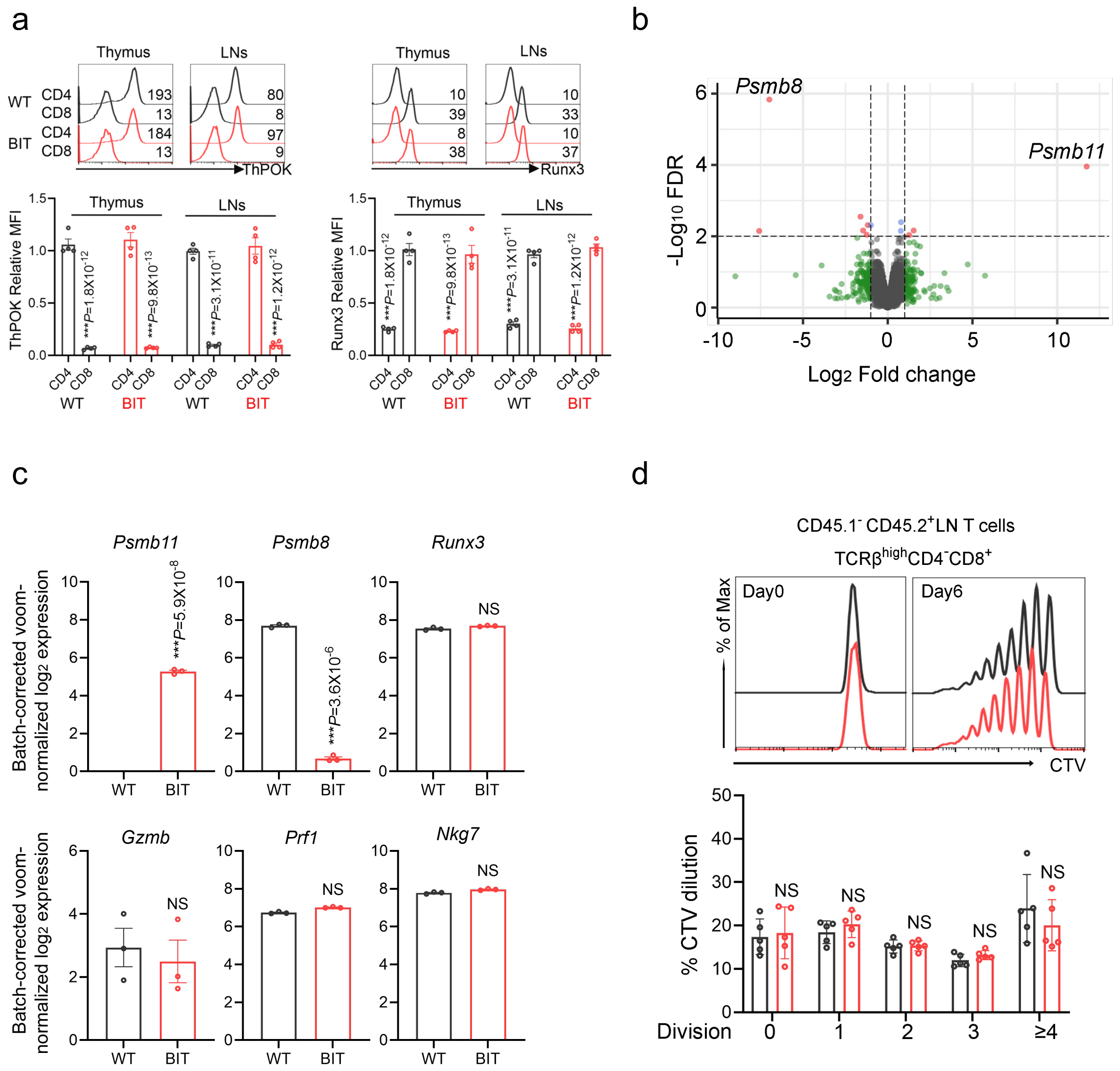

Supplementary Figure 9. Transcriptomic and functional analyses of CD8^{+} T cells generated in BIT mice.

a. Flow cytometric analysis of ThPOK (left) and Runx3 (right) expression in CD4^{+}CD8^{-} TCRβ^{high} cells (CD4) and CD4^{-}CD8^{+} TCRβ^{high} cells (CD8) from the thymus and the lymph nodes (LNs) of WT (n=4) and BIT (n=4) mice . Representative flow cytometric profiles (top) and relative mean fluorescence intensities (MFIs) (bottom) are shown. Numbers in flow cytometric profiles indicate MFI values. Results indicate the equivalent and selective expression of ThPOK and Runx3 in CD4 and CD8 T cells, respectively, isolated from WT and BIT mice.

b. Volcano plot of RNA sequencing analysis of CD4^{-}CD8^{+} TCRβ^{high} T cells isolated from the LNs of WT and BIT mice. Note that *Psmb11* was ectopically expressed instead of *Psmb8* in BIT CD8 T cells. Among 13,674 genes detected in the analysis, only two genes (*Scart2* and *Bbc3*) and five genes (*Rell1*, *Mid1*, *Slc28a2b*, *5830416l19Rik*, and *H2ac24*) other than *Psmb11* and *Psmb8* were higher (> 2-fold, P < 0.01) in the expression in BIT and WT CD8 T cells, respectively (red dots).

c. Relative expression of indicated genes from RNA sequencing analysis of CD4^{-}CD8^{+} TCRβ^{high} T cells isolated from the LNs of WT (n=3) and BIT (n=3) mice. *Gzmb*, granzyme B; *Prf1*, perforin 1; *Nkg7*, natural killer cell granule protein 7. Dots represent the data from individual mice. Statistical analysis was performed using an unpaired two-tailed t-test. ***P < 0.001; NS, not significant.

d. Cell Trace Violet (CTV) dilution assay for *in vivo* T cell proliferation. CTV-labelled T cells (CD45.1^{-}CD45.2^{+}) were injected intravenously into sublethally irradiated host B6 mice (CD45.1^{+}CD45.2^{-}). Cells were analyzed at day 0 and day 6 after injection (top). Frequencies of cells with indicated numbers of cell division are plotted. Dots represent the data from individual mice (WT, n=5; BIT, n=5). Data are representative of three independent experiments. Statistical analysis was performed using an unpaired two-tailed t-test. NS, not significant.

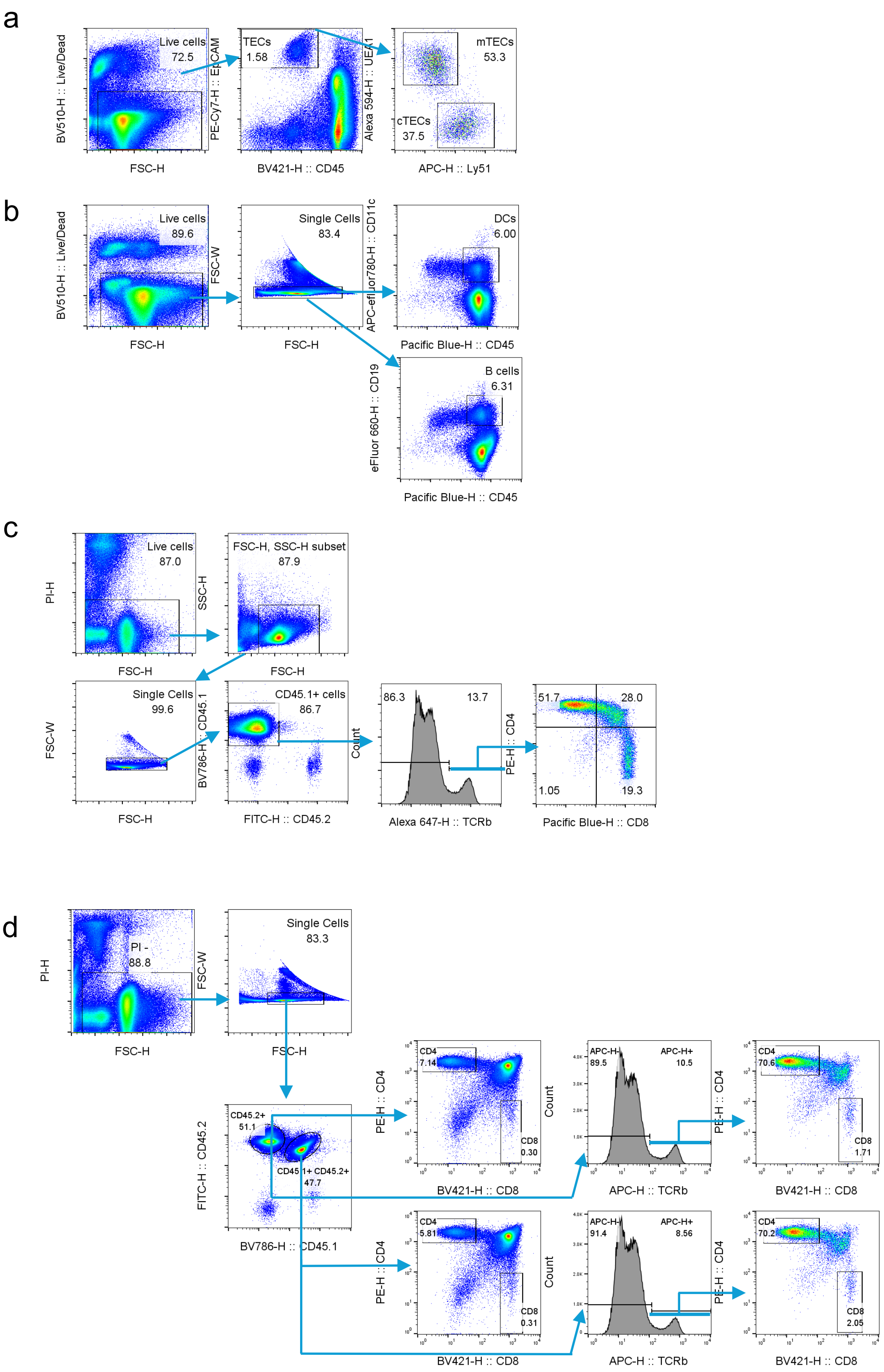

Supplementary Figure 10. Flow cytometric gating strategies.

a. Gating strategy for thymic epithelial cells (TECs) used in Fig. 1c and S2a-d. Viable cells (FSC-H, PI^{-}) gated for CD45^{low} EpCAM^{high} TECs were further gated for Ly51^{+}UEA1^{-} cortical TECs (cTECs) and Ly51^{-}UEA1^{+} medullary TECs (mTECs).

b. Gating strategy for dendritic cells (DCs) and B cells used in Fig. 1c, S2a-d, and S8. Viable singlet cells (FSC-H, PI^{-}; FSC-H vs FSC-W) were gated for CD45^{high} CD11c^{high} DCs and CD45^{high} CD19^{high} B cells.

c. Gating strategy for bone marrow chimera experiments used in Fig. 3a-d.

d. Gating strategy for mixed bone marrow chimera experiments used in Fig. 4a-c and 7a, b.

Numbers indicate the frequency of cells in indicated box.

717	CTTCCTTGTCGCAGAGTCGA**CGG**
718*	GCAGAGTCGACGGATCTTCG**GGG**
719	GACGGATCTTCGGGGCCAAG**TGG**
720	GATCTGTGCGGTGCCGCTCG**GGG**
721	CTCGGGCCGCTGCCCCCGAG**CGG**
722	TCGGGGGCAGCGGCCCGAGT**GGG**
723	TTTACTACGTAGATGACAAT**GGG**
724	CGGGAACACCTATGCCTATG**GGG**
725	GATGGACAGTGGTTACCGGC**AGG**
726	AGCTCTGCGGCCAAGGTCGT**AGG**
727*	AGCTTAGATTGGGAGCCTCG**GGG**
728	GCTTAGATTGGGAGCCTCGG**GGG**
759*	TCCGGAGCTCGCACTTCCCC**GGG**
760	CCGGAGCTCGCACTTCCCCG**GGG**

Supplementary Table 1. Guide RNAs designed and used for mouse genome editing.
*used for targeting experiments.

Item	Labeled fluorescence	clone	Vender	Catalog number	PRID	Working dilution
AIRE	eFluor™ 660	5H12	Invitrogen	50-5934-82	RRID:AB_2574257	1:100
Goat anti-Rabbit IgG (H+L)	Alex555		Invitrogen	A21428	RRID:AB_141784	1:400
H-2D$_b$	PE	28-14-8	eBioscience	12-5999-82	RRID:AB_466125	1:100
H-2K$_b$	Alex647	AF6-88.5	Biolegend	116512	RRID:AB_492917	1:100
H-2K$_b$	BV786	AF6-88.5	BD Bioscience	742863	RRID:AB_2741105	1:100
H-2K$_b$	FITC	AF6-88.5	BD Bioscience	562002	RRID:AB_10924590	1:100
B220	PE	RA3-6B2	BD bioscience	553090	RRID:AB_394619	1:100
β-Actin		2A3	Santacruz	sc-517582 HRP		1:100
CCR7	Alex647	4B12	R&D System	FAB3477R-100UG	RRID:AB_3649367	1:100
CD11c	Alex647	N418	Biolegend	117312	RRID:AB_389328	1:100
CD11c	APC-eFluor780	N418	eBioscience	47-0114-B2	RRID:AB_1548663	1:100
CD19	eFluor™ 660	1D3	eBioscience	50-0193-82	RRID:AB_11218286	1:100
CD249(Ly51)	BV421	6C3	BD Bioscience	740013	RRID:AB_2739785	1:200
CD3		17A2	eBioscience	14-0032-82	RRID:AB_467053	1:100
CD326 (EpCAM)	PE/Cy7	G8.8	Biolegend	118216	RRID:AB_1236471	1:200
CD326 (EpCAM)	BV711	G8.8	Biolegend	118233	RRID:AB_2632775	1:200
CD4	eFluor 780	RM4-5	Invitrogen	47-0042-82	RRID:AB_1272183	1:100
CD4	PE/Cy7	RM4-5	Biolegend	100528	RRID:AB_312729	1:100
CD4	PE	RM4-5	Biolegend	100512	RRID:AB_312714	1:100
CD45	BV421	30-F11	Biolegend	103134	RRID:AB_2562559	1:40
CD45	PE/Cy7	30-F11	Biolegend	103114	RRID:AB_312979	1:40
CD45.1	BV786	A20	BD Bioscience	740889	RRID:AB_2740538	1:100
CD45.2	FITC	104	BD Pharmigen	553722	RRID:AB_10829797	1:100
CD69	BV786	H1.2F3	BD Bioscience	564683	RRID:AB_2738890	1:100
CD69	PE	H1.2F3	Biolegend	104507	RRID:AB_313111	1:100
CD8a	PB	53-6.7	Biolegend	100725	RRID:AB_493425	1:100
Active Caspase-3	PE	C92-605.rMAb	BD pharmigen	570185	RRID:AB_3685574	1:100
Goat anti-Rabbit IgG (H+L)			Invitrogen	31460	RRID:AB_228341	1:2500
Ly51	Alex647	6C3	Biolegend	108312	RRID:AB_2099614	1:200
PSMB8/LMP7		D1K7X	Cell Signaling	13635S	RRID:AB_2744693	1:1000
RUNX3	PE	R3-5G4	BD Bioscience	564814	RRID:AB_2738969	1:25
TCRb	Alex647	H57-597	Biolegend	109218	RRID:AB_493346	1:50
TCRb	Alex594	H57-597	Biolegend	109238	RRID:AB_2563324	1:50
TCRb	FITC	H57-597	Biolegend	109205	RRID:AB_313428	1:50
UEA1	DyLight® 594		Vector Laboratories	DL-1067-1		1:200
Zbtb7b(Thpok)	Alex647	T43-94	BD Bioscience	565500	RRID:AB_2739268	1:50
β5t		CPTC-PSMB11(mouse)-1	Antibody Characterization Program of the National Cancer Institute	https://antibodies.cancer.gov/browse		1:1000
CD45 MicroBeads, mouse			Miltenyi Biotec	130-052-301		

Supplementary Table 2. Antibodies and reagents used in this study
