# Supplementary material for: Proteasome alteration between epithelial and hematopoietic cells facilitates positive selection of CD8 T cells
Source: Nat Commun. 2026 Apr 27;17:5780. doi: 10.1038/s41467-026-72411-x (PMC13324746; doi:10.1038/s41467-026-72411-x)
Supplement: Supplementary file 2 — Reporting Summary [file 41467_2026_72411_MOESM2_ESM.pdf]

nature portfolio

Corresponding author(s): Yousuke Takahama

Last updated by author(s): 4/1/2026

Reporting Summary

Nature Portfolio wishes to improve the reproducibility of the work that we publish. This form provides structure for consistency and transparency in reporting. For further information on Nature Portfolio policies, see our Editorial Policies and the Editorial Policy Checklist.

Statistics

For all statistical analyses, confirm that the following items are present in the figure legend, table legend, main text, or Methods section.

n/a	Confirmed	
	☒	The exact sample size (*n*) for each experimental group/condition, given as a discrete number and unit of measurement
	☒	A statement on whether measurements were taken from distinct samples or whether the same sample was measured repeatedly
	☒	The statistical test(s) used AND whether they are one- or two-sided<br>*Only common tests should be described solely by name; describe more complex techniques in the Methods section.*
☒		A description of all covariates tested
	☒	A description of any assumptions or corrections, such as tests of normality and adjustment for multiple comparisons
	☒	A full description of the statistical parameters including central tendency (e.g. means) or other basic estimates (e.g. regression coefficient) AND variation (e.g. standard deviation) or associated estimates of uncertainty (e.g. confidence intervals)
	☒	For null hypothesis testing, the test statistic (e.g. *F*, *t*, *r*) with confidence intervals, effect sizes, degrees of freedom and *P* value noted<br>*Give P values as exact values whenever suitable.*
☒		For Bayesian analysis, information on the choice of priors and Markov chain Monte Carlo settings
☒		For hierarchical and complex designs, identification of the appropriate level for tests and full reporting of outcomes
☒		Estimates of effect sizes (e.g. Cohen's *d*, Pearson's *r*), indicating how they were calculated

Our web collection on statistics for biologists contains articles on many of the points above.

Software and code

Policy information about availability of computer code

Data collection	LSRII, FACS Fortessa, FACSAriaSORP (BD Bioscience),<br>Nikon Eclipse Ti2 microscope (Nikon), CSU-W1 Spinning disk scanner (Yokogawa) with Hamamatsu Orca Flash 4.0 camera, NIS-Elements software (Nikon), QuantStudio 6 Flex Real-time PCR System (Applied Biosystems), NextSeq 2000 (Illumina).
Data analysis	FlowJo version v10.6.2, Prism 10 (Graph pad software), ImageJ version 1.53o (NIH)

For manuscripts utilizing custom algorithms or software that are central to the research but not yet described in published literature, software must be made available to editors and reviewers. We strongly encourage code deposition in a community repository (e.g. GitHub). See the Nature Portfolio guidelines for submitting code & software for further information.

Data

Policy information about availability of data

All manuscripts must include a data availability statement. This statement should provide the following information, where applicable:

- Accession codes, unique identifiers, or web links for publicly available datasets
- A description of any restrictions on data availability
- For clinical datasets or third party data, please ensure that the statement adheres to our policy

Bulk RNA-sequencing data (GEO accession ID: GSE319325) and TCR sequencing data (GEO accession ID: GSE322772)

Research involving human participants, their data, or biological material

Policy information about studies with human participants or human data. See also policy information about sex, gender (identity/presentation), and sexual orientation and race, ethnicity and racism.

Reporting on sex and gender	N/A
Reporting on race, ethnicity, or other socially relevant groupings	N/A
Population characteristics	N/A
Recruitment	N/A
Ethics oversight	N/A

Note that full information on the approval of the study protocol must also be provided in the manuscript.

Field-specific reporting

Please select the one below that is the best fit for your research. If you are not sure, read the appropriate sections before making your selection.

☒ Life sciences ☐ Behavioural & social sciences ☐ Ecological, evolutionary & environmental sciences

For a reference copy of the document with all sections, see nature.com/documents/nr-reporting-summary-flat.pdf

Life sciences study design

All studies must disclose on these points even when the disclosure is negative.

Sample size	Sample size for each experiment is indicated in figure legends. At least two independent experiments with three replicates were conducted as the minimum sample size.
Data exclusions	Data were excluded when samples failed quality control, such as abnormal FACS profiles (poor separation) or when animals exhibited signs of illness prior to sample collection.
Replication	For all experiments, at least three replicates were analyzed in at least two independent experiments. The experimental findings were reliably reproduced.
Randomization	Animals were allocated to groups based on their genotypes.
Blinding	No blinding was used.

Reporting for specific materials, systems and methods

We require information from authors about some types of materials, experimental systems and methods used in many studies. Here, indicate whether each material, system or method listed is relevant to your study. If you are not sure if a list item applies to your research, read the appropriate section before selecting a response.

Materials & experimental systems

n/a	Involved in the study	
☐	☒	Antibodies
☒	☐	Eukaryotic cell lines
☒	☐	Palaeontology and archaeology
☐	☒	Animals and other organisms
☒	☐	Clinical data
☒	☐	Dual use research of concern
☒	☐	Plants

Methods

n/a	Involved in the study	
☒	☐	ChIP-seq
☐	☒	Flow cytometry
☒	☐	MRI-based neuroimaging

Antibodies

Antibodies used	AIRE eFluor™ 660 5H12 Invitrogen 50-5934-82 RRID:AB_2574257 1:100<br>Goat anti-Rabbit IgG (H+L) Alex555 Invitrogen A21428 RRID:AB_141784 1:400<br>H-2Db PE 28-14-8 eBioscience 12-5999-82 RRID:AB_466125 1:100<br>H-2Kb Alex647 AF6-88.5 Biolegend 116512 RRID:AB_492917 1:100

H-2Kb BV786 AF6-88.5 BD Bioscience 742863 RRID:AB_2741105 1:100
H-2Kb FITC AF6-88.5 BD Bioscience 562002 RRID:AB_10924590 1:100
B220 PE RA3-6B2 BD bioscience 553090 RRID:AB_394619 1:100
β-Actin 2A3 Santacruz sc-517582 HRP 1:100
CCR7 Alex647 4B12 R&D System FAB3477R-100UG RRID:AB_3649367
CD11c Alex647 N418 Biolegend 117312 RRID:AB_389328 1:100
CD11c APC-eFluor780 N418 eBioscience 47-0114-B2 RRID:AB_1548663 1:100
CD19 eFluor™ 660 1D3 eBioscience 50-0193-82 RRID:AB_11218286 1:100
CD249(Ly51) BV421 6C3 BD Bioscience 740013 RRID:AB_2739785 1:200
CD3 17A2 eBioscience 14-0032-82 RRID:AB_467053 1:100
CD326 (EpCAM) PE/Cy7 G8.8 Biolegend 118216 RRID:AB_1236471 1:200
CD326 (EpCAM) BV711 G8.8 Biolegend 118233 RRID:AB_2632775 1:200
CD4 eFluor 780 RM4-5 Invitrogen 47-0042-82 RRID:AB_1272183 1:100
CD4 PE/Cy7 RM4-5 Biolegend 100528 RRID:AB_312729 1:100
CD4 PE RM4-5 Biolegend 100512 RRID:AB_312714 1:100
CD45 BV421 30-F11 Biolegend 103134 RRID:AB_2562559 1:40
CD45 PE/Cy7 30-F11 Biolegend 103114 RRID:AB_312979 1:40
CD45.1 BV786 A20 BD Bioscience 740889 RRID:AB_2740538 1:100
CD45.2 FITC 104 BD Pharmigen 553722 RRID:AB_10829797 1:100
CD69 BV786 H1.2F3 BD Bioscience 564683 RRID:AB_2738890 1:100
CD69 PE H1.2F3 Biolegend 104507 RRID:AB_313111 1:100
CD8a PB 53-6.7 Biolegend 100725 RRID:AB_493425 1:100
Active Caspase-3 PE C92-605.rMAb BD pharmigen 570185 RRID:AB_3685574 1:100
Goat anti-Rabbit IgG (H+L) Invitrogen 31460 RRID:AB_228341 1:2500
Ly51 Alex647 6C3 Biolegend 108312 RRID:AB_2099614 1:200
PSMB8/LMP7 D1K7X Cell Signaling 13635S RRID:AB_2744693 1:1000
RUNX3 PE R3-5G4 BD Bioscience 564814 RRID:AB_2738969 1:25
TCRb Alex647 H57-597 Biolegend 109218 RRID:AB_493346 1:50
TCRb Alex594 H57-597 Biolegend 109238 RRID:AB_2563324 1:50
TCRb FITC H57-597 Biolegend 109205 RRID:AB_313428 1:50
UEA1 DyLight® 594 Vector Laboratories DL-1067-1 1:200
Zbtb7b(Thpok) Alex647 T43-94 BD Bioscience 565500 RRID:AB_2739268 1:50
β5t (CPTC-PSMB11(mouse)-1 Antibody Characterization Program of the National Cancer Institute https://antibodies.cancer.gov/browse 1:1000

Validation

All antibodies are commercially available and have been validated by the manufacture or in previous reports.

Animals and other research organisms

Policy information about studies involving animals; ARRIVE guidelines recommended for reporting animal research, and Sex and Gender in Research

Laboratory animals

C57BL/6 (B6) mice and B6-CD45.1 (B6.SJL-PtprcaPtpcb) mice were obtained from The Jackson Laboratory. B6 mice were also obtained from Charles River Laboratories. beta5t-deficient mice, beta5i-deficient mice, TCRalpha-deficient mice, Rag1-deficient mice, beta2m-deficient mice, I-Ab-deficient mice, Rosa26INDIA knock-in mice, pLck-Bcl2-transgenic mice were previously described. All mice were backcrossed to B6 background and maintained in our animal facility.

Wild animals

No wild animals were used in this study.

Reporting on sex

Both male and female mice were used and analyzed at age 2-12 weeks old.

Field-collected samples

No field-collected samples were used in this study.

Ethics oversight

All animal experiments were approved by the National Cancer Institute Animal Care and Use Committee. All animals were maintained in accordance with US National Institutes of Health guidelines.

Note that full information on the approval of the study protocol must also be provided in the manuscript.

Plants

Seed stocks	N/A
Novel plant genotypes	N/A
Authentication	N/A

Flow Cytometry

Plots

Confirm that:

- [ ] The axis labels state the marker and fluorochrome used (e.g. CD4-FITC).
- [x] The axis scales are clearly visible. Include numbers along axes only for bottom left plot of group (a 'group' is an analysis of identical markers).
- [x] All plots are contour plots with outliers or pseudocolor plots.
- [x] A numerical value for number of cells or percentage (with statistics) is provided.

Methodology

Sample preparation	Single cell suspensions were prepared by gently tweezing the organs with forceps in cold HBSS supplemented with 0.5% BSA and 0.5% NaN3.
Instrument	LSRII, Fortessa, FACSAria (BD Biosciences)
Software	FlowJo version v10.6.2
Cell population abundance	>95% on sorted cells, which was determined by flow cytometry analysis on post sorted cells.
Gating strategy	Live cells were defined by FSC/SSC gating and staining with propidium iodide or Ghost Dye Violet 510 LIVE/DEAD (Tonbo Biosciences) for fresh and fixed staining, respectively. All gating strategies are stated in the manuscript.

- [x] Tick this box to confirm that a figure exemplifying the gating strategy is provided in the Supplementary Information.
