# Supplementary material for: Proteasome alteration between epithelial and hematopoietic cells facilitates positive selection of CD8 T cells
Source: Nat Commun. 2026 Apr 27;17:5780. doi: 10.1038/s41467-026-72411-x (PMC13324746; doi:10.1038/s41467-026-72411-x)
Supplement: Supplementary file 3 — Transparent Peer Review File [file 41467_2026_72411_MOESM3_ESM.pdf]

nature portfolio

Peer Review File

Proteasome alteration between epithelial and hematopoietic cells facilitates positive selection of CD8 T cells

Corresponding Author: Professor Yousuke Takahama

This manuscript has been previously reviewed at another journal. This document only contains information relating to versions considered at Nature Communications.

This file contains all reviewer reports in order by version, followed by all author rebuttals in order by version.

Version 0:

Reviewer comments:

Reviewer #2

(Remarks to the Author)
The authors have carefully responded to the comments. The new data in Fig 7f are useful showing how early the BIT hindrance can be detected. Results and interpretation for Fig 7a,b are now described more clearly. Similarly, the main take-away point and novel hypothesis is better explained ("....contribution of previously unknown mechanisms in securing the unique capability of cTECs, rather than other cortical APCs, to induce positive selection in the thymic cortex.").

Reviewer #3

(Remarks to the Author)
Matsuda-Lennikov et al modulate b5t expression in distinct subsets to test the prevailing view that the absence of b5t from the medulla prevents developing T cells that are reactive to b5t-dependent peptides from being rejected in the medulla. The key finding is that ectopic expression of b5t in medullary epithelial cells was inconsequential; however, its ectopic expression in hematopoietic cells attenuated CD8 T cell development by disrupting cortical positive selection at a relatively early stage (CD69+CCR7-). These findings are inconsistent with the current paradigm of the cTEC to mTEC proteosome switch, and that the critical difference in proteosomes is that between cTEC and hematopoietic progenitors, specifically nonB/nonT cells, and is mediated in a cell-extrinsic manner. Finally, the ectopic expression of b5t in hematopoietic cells alter the TCR repertoire in ways that are partially distinct from those occurring in b5t-deficiency.
These studies employ elegant models and experiments that are well conceived and executed that are thoughtfully interpreted. Moreover, the findings of this study make an important contribution as they clearly contradict the prevailing view in favor of the proteosome switch model.

Reviewer #4

(Remarks to the Author)
The authors have fully addressed all the concerns raised by the reviewers, and their work provides new insights as to the role and requirement for a specialized set of peptides generated by the thymoproteosome (b5t+) presented by cortical thymus epithelial cells (cTEC) for the selection and lineage commitment of CD8 T cells. Their work nicely extends the recent publication from the Singer lab in Nature Immunology, and in essence adds a key experimental model system that was missing in that work, which is the expression of the thymoproteosome in hematopoietic antigen presenting cells (APC), and interrogate the effect that this would have on CD8 selection, given the constraints specified by the Kinetic Signaling model. Overall the work is extremely timely and rigorously performed and crisply analyzed, it is also a very ingeniously conceived genetic approach, which will likely yield additional insights in future experimental approaches.

Super minor points:

1- On line 45, the use of the word "preselected" is misleading, as this would typically mean that the germline repertoire has been PRE-selected, i.e., already selected, which is not what the authors mean, rather the meaning should be that is has yet

to be selected, and preselected does not convey that point. Perhaps the authors should state it as non-selected or unselected. This misuse is also present in line 284.

2- In line 427, when the authors point out to the possibility that the alternative lineage T cells could be selected for in the BIT mice, it would be good to have them reflect on the recent paper from the Singer lab, and mention whether innate memory (IM) CD8 T cells could be one of these alternative lineages. In particular, as the authors did not fully characterize for those outcomes, by looking for changes in Eomes expression or by using mouse strains that are more likely to yield those outcomes, eg., a Balb/c background. Please note, I am not asking for experiments, just a comment on this possibility in the discussion section.

Open Access This Peer Review File is licensed under a Creative Commons Attribution 4.0 International License, which permits use, sharing, adaptation, distribution and reproduction in any medium or format, as long as you give appropriate credit to the original author(s) and the source, provide a link to the Creative Commons license, and indicate if changes were made.

In cases where reviewers are anonymous, credit should be given to 'Anonymous Referee' and the source.

The images or other third party material in this Peer Review File are included in the article’s Creative Commons license, unless indicated otherwise in a credit line to the material. If material is not included in the article’s Creative Commons license and your intended use is not permitted by statutory regulation or exceeds the permitted use, you will need to obtain permission directly from the copyright holder.

To view a copy of this license, visit https://creativecommons.org/licenses/by/4.0/

Point-by-point response to reviewers' comments

Reviewer #2:
The authors have carefully responded to the comments. The new data in Fig 7f are useful showing how early the BIT hindrance can be detected. Results and interpretation for Fig 7a,b are now described more clearly. Similarly, the main take-away point and novel hypothesis is better explained ("....contribution of previously unknown mechanisms in securing the unique capability of cTECs, rather than other cortical APCs, to induce positive selection in the thymic cortex.").
Thank you for the positive comments.

Reviewer #3:
Matsuda-Lennikov et al modulate b5t expression in distinct subsets to test the prevailing view that the absence of b5t from the medulla prevents developing T cells that are reactive to b5t-dependent peptides from being rejected in the medulla. The key finding is that ectopic expression of b5t in medullary epithelial cells was inconsequential; however, its ectopic expression in hematopoietic cells attenuated CD8 T cell development by disrupting cortical positive selection at a relatively early stage (CD69+CCR7-). These findings are inconsistent with the current paradigm of the cTEC to mTEC proteosome switch, and that the critical difference in proteosomes is that between cTEC and hematopoietic progenitors, specifically nonB/nonT cells, and is mediated in a cell-extrinsic manner. Finally, the ectopic expression of b5t in hematopoietic cells alter the TCR repertoire in ways that are partially distinct from those occurring in b5t-deficiency.
These studies employ elegant models and experiments that are well conceived and executed that are thoughtfully interpreted. Moreover, the findings of this study make an important contribution as they clearly contradict the prevailing view in favor of the proteosome switch model.
Thank you for the supporting comments.

Reviewer #4:
The authors have fully addressed all the concerns raised by the reviewers, and their work provides new insights as to the role and requirement for a specialized set of peptides generated by the thymoproteosome (b5t+) presented by cortical thymus epithelial cells (cTEC) for the selection and lineage commitment of CD8 T cells. Their work nicely extends the recent publication from the Singer lab in Nature Immunology, and in essence adds a key experimental model system that was missing in that work, which is the expression of the thymoproteosome in hematopoietic antigen presenting cells (APC), and interrogate the effect that this would have on CD8 selection, given the constraints specified by the Kinetic Signaling model. Overall the work is extremely timely and rigorously performed and crisply analyzed, it is also a very ingeniously conceived genetic approach, which will likely yield additional insights in future experimental approaches.
Thank you for the favorable comments.

Super minor points:
1- On line 45, the use of the word "preselected" is misleading, as this would typically mean that the germline repertoire has been PRE-selected, i.e., already selected, which is not what the authors mean, rather the meaning should be that is has yet to be selected, and preselected does not convey that point.

Perhaps the authors should state it as non-selected or unselected. This misuse is also present in line 284.

The text was revised according to the comments (pages 3 and 14).

2- In line 427, when the authors point out to the possibility that the alternative lineage T cells could be selected for in the BIT mice, it would be good to have them reflect on the recent paper from the Singer lab, and mention whether innate memory (IM) CD8 T cells could be one of these alternative lineages. In particular, as the authors did not fully characterize for those outcomes, by looking for changes in Eomes expression or by using mouse strains that are more likely to yield those outcomes, eg., a Balb/c background. Please note, I am not asking for experiments, just a comment on this possibility in the discussion section.

The text was revised with the additional reference, according to the comments (page 20 and new reference 42).
